# Combining sterile and incompatible insect techniques for the population suppression of *Drosophila suzukii*

**DOI:** 10.1007/s10340-020-01199-6

**Published:** 2020-01-29

**Authors:** K. Nikolouli, F. Sassù, L. Mouton, C. Stauffer, K. Bourtzis

**Affiliations:** 1 Department of Forest and Soil Sciences, Boku, University of Natural Resources and Life Sciences, Vienna, Austria; 2 Insect Pest Control Section, Joint FAO/IAEA Division of Nuclear Techniques in Food and Agriculture, Wagramerstrasse 5, PO Box 100, 1400 Vienna, Austria; 3 Laboratoire de Biométrie et Biologie Evolutive, CNRS, Université de Lyon, Université Claude Bernard Lyon 1, 69100 Villeurbanne, France

**Keywords:** *Wolbachia*, Spotted wing *Drosophila*, Biological control, Area-wide integrated pest management

## Abstract

The spotted wing *Drosophila*, *Drosophila suzukii*, has recently invaded Europe and the Americas, and it is a major threat for a wide variety of commercial soft fruits both in open field and greenhouse production systems. *D. suzukii* infests a wide range of ripening fruits, leading to substantial yield and revenue losses. As the application of insecticides close to the harvest period poses great concerns, the development of an efficient environment-friendly control approach to fight *D. suzukii* is necessary. In this study, we exploited the sterile insect technique (SIT) in combination with *Wolbachia* symbiosis as a population suppression approach that can constitute a potential component of an area-wide integrated pest management program. We aimed to establish a combined SIT/incompatible insect technique (IIT) protocol that would require lower irradiation doses as a complementary tool for *D. suzukii* management. Two *D. suzukii* lines trans-infected with the *Wolbachia w*Ha and *w*Tei strains were irradiated at doses four times less than usual (e.g., 45 Gy), and the egg hatching and adult emergence were determined. Our results indicated that *w*Ha and *w*Tei females as well as *w*Ha males were sterile at this low dose. The longevity, adult emergence and flight ability of adults were evaluated, and no major effect caused by irradiation was detected. Our data indicate that a SIT/IIT protocol can be a competent approach for *D. suzukii* management.

**Electronic supplementary material:**

The online version of this article (10.1007/s10340-020-01199-6) contains supplementary material, which is available to authorized users.

## Key message

- *Wolbachia*-trans-infected *D. suzukii* lines were irradiated with 45–60–90 Gy irradiation doses, and egg hatch rate and F1 progeny production were significantly affected.
- Longevity emergence rate and flight ability were not affected after irradiation at 45 Gy.
- A combined SIT/IIT strategy is proposed for the population suppression of *D. suzukii* populations as part of an area-wide integrated pest management program.

## Introduction

The spotted wing *Drosophila* (SWD), *Drosophila suzukii* (Matsumura) (Diptera: Drosophilidae), is an invasive species originated from Asia that has been recognized as a major concern in agriculture since its first confirmed records in Europe and North America in 2008 (Calabria et al. 2012; Hauser 2011). Predictive models indicate that Australia and Africa offer suitable environmental conditions for potential future invasion of the species (Dos Santos et al. 2017). The constant invasion in new areas has rapidly escalated this pest into a significant global threat for several commercial fruit crops (Asplen et al. 2015; Bolda et al. 2010; Calabria et al. 2012; Cini et al. 2012; Deprá et al. 2014; Walsh et al. 2011). Although different invasion routes have been reported for the European and North American populations, the detection of *D. suzukii* in these two continents was simultaneous, thus supporting the notion that worldwide trade practices are its main dispersal source (Fraimout et al. 2017).

Unique morphological and biological traits of *D. suzukii*, including the female’s serrated ovipositor, the short generation time and the ability to adapt in wide temperature and humidity ranges, advanced the dispersal potential and establishment of this pest globally (Hamby et al. 2016; Lee et al. 2011; Sánchez-Ramos et al. 2019a, b; Tochen et al. 2014, 2016; Wong et al. 2018). A series of soft-skinned fruits, both cultivated and wild, serves as trophic niches and oviposition substrates for *D. suzukii,* and this polyphagy behavior is a key factor for the invasion success of the fly (Lee et al. 2011; Poyet et al. 2015). The high availability of soft fruit crops and their different ripening times throughout the year, combined with the presence of wild hosts, assisted not only in the invasion process, but also in the exceptionally fast adaptation of the fly in the new habitats (Cini et al. 2012; Poyet et al. 2015; Rota-Stabelli et al. 2013; Santoiemma et al. 2019; Tait et al. 2018). As a result the economic impact in the agricultural areas and fruit industry has been enormous (Bolda et al. 2010; De Ros et al. 2015; Mazzi et al. 2017). Extended yield and revenue losses have been reported, while the monitoring and management investments are an additive factor in the economic implications that farmers and companies need to face (DiGiacomo et al. 2019; Goodhue et al. 2011; Mazzi et al. 2017).

Insecticide applications are currently the front-line method used to control *D. suzukii* in conventional and organic crop areas (Beers et al. 2011; Bruck et al. 2011; Grassi et al. 2011; Rota-Stabelli et al. 2013; Sial et al. 2019; Van Timmeren and Isaacs 2013). Application of insecticides close to the harvest period poses great concerns for the health of both farmers and consumers (Rota-Stabelli et al. 2013), but also to beneficial arthropods (Desneux et al. 2007). The total number of applications for most insecticides is restricted by regulation but, on the other hand, the short generation time of *D. suzukii* requires frequent applications when fruits are at the ripening stage which needs chemicals with a shorter pre-harvest interval (Cini et al. 2012; Goodhue et al. 2011; Sial et al. 2019). This can result in increased insecticide residuals in fruits and unpredictable impacts on pollinators and other non-target species, including *D. suzukii*’s natural enemies (Iglesias and Liburd 2017; Rota-Stabelli et al. 2013; Roubos et al. 2014; Walsh et al. 2011).

The regulatory limitations governing the classical chemical control methods coupled with the concerns over the risks of their use demonstrate the urgent need to develop an alternative environmentally sound and sustainable method to combat *D. suzukii* (Cuthbertson et al. 2014; Haye et al. 2016; Nikolouli et al. 2018). Insecticide pest resistance, residuals in food, environmental contamination, outbreaks of secondary pests, and reductions in populations of beneficial insects are only some of the major environmental problems that had been caused by the indiscriminate use of insecticides (Bourtzis et al. 2016). The increasing worldwide demand for pest control methods that are both efficient and friendly to the environment has advanced the sterile insect technique (SIT)-based programs. The SIT is a species-specific method of pest population control that relies on mass-produced insects (only males, if feasible) which are sterilized with ionizing radiation, transferred in the target area and bulk-released until an overflow ratio has been created (Dyck et al. 2005). The success of the SIT relies on the competence of the sterile males to mate with the wild fertile females leading eventually to substantial progeny decline (Dyck et al. 2005; Knipling 1979). The SIT has been for years the workhorse of a plethora of area-wide integrated pest management (AW-IPM) programs since its first application against the New World screwworm fly in the 1950’s (Dyck et al. 2005; Vreysen et al. 2006). Ever since, the SIT has been successfully launched worldwide to combat various fruit fly species of economic importance and disease vectors of livestock and humans (Cayol et al. 2002; Munhenga et al. 2016; Pereira et al. 2013; Vreysen et al. 2006).

The performance and competitiveness of the released sterile males are important quality parameters and, if not adequate, they might seal the fate of a SIT program (Calkins and Parker 2005). The irradiation dose required for complete male sterility might have profound implications for the biological quality and male mating competitiveness of the insect, as demonstrated for the fruit flies *Ceratitis capitata*, *Anastrepha ludens* and *Anastrepha obliqua* (Guerfali et al. 2012; Rull et al. 2007; Toledo et al. 2004). Significant knowledge has been gained from SIT applications in fruit flies and invested in the development of a biocontrol method against *D. suzukii* with a SIT component. Lanouette et al. (2017), Krüger et al. (2018) and Sassù et al. (2019) (defined the irradiation doses that sterilize males and females without affecting the emergence, longevity and flight ability of the flies.

As shown for the mosquito vector species *Aedes albopictus*, the combination of the SIT with the IIT could be a well-suited approach for *D. suzukii* management, as an alternative method to the irradiation-induced sterility (Nikolouli et al. 2018; Zhang et al. 2015b). The IIT employs the mechanism of cytoplasmic incompatibility (CI) in order to produce conditionally sterile males for the control of insect pest populations and disease vectors. CI induced by symbiotic bacteria such as *Wolbachia* has been studied thoroughly for its potential as a pest population control strategy and has already been exploited against mosquito species in semi-field and field trials (Atyame et al. 2015; Laven 1967; Mains et al. 2019; O’Connor et al. 2012). However, natural *Wolbachia* infections do not occur in all insect species or may not induce CI in their hosts. Trans-infections using embryo microinjections permit host infections with exogenous *Wolbachia* strains capable of inducing CI (Hughes and Rasgon 2014; Zabalou et al. 2004). Following the proven record of success in tephritid fruit flies (Apostolaki et al. 2011; Zabalou et al. 2004), Cattel and his colleagues (2018) applied this trans-infection approach in *D. suzukii*. Previous studies reported that the natural *Wolbachia* infection in *D. suzukii* (*w*Suz) shows a variable infection frequency and it is not able to induce strong CI in its host (Cattel et al. 2016; Hamm et al. 2014). Therefore, two *Wolbachia* strains (*w*Ha and *w*Tei) acquired by other *Drosophila* species were microinjected into *D. suzukii* embryos and stable trans-infected lines were established in the laboratory. In both lines, *Wolbachia* induced strong CI (95.57% and 96.46%, respectively) that was not rescued by the *w*Suz strain thus giving rise in two promising candidates for a combined SIT/IIT strategy in *D. suzukii* (Cattel et al. 2018; Nikolouli et al. 2018). The mechanism of this approach has been previously dissected against mosquito vectors (Bourtzis et al. 2016; Lees et al. 2015; Zhang et al. 2015a, b, 2016; Zheng et al. 2019), and it can bear promising results if a low radiation dose to ensure female sterility is applied in flies infected with a *Wolbachia* strain that induces CI. In such a system, bisexual releases would be feasible since the *Wolbachia*-infected females would be sterile and the risk of population replacement would be avoided (Lees et al. 2015; Zhang et al. 2015b). In addition, the released males would be able to introduce sterility in the wild population through the combined action of radiation and CI.

In the present study, we aimed to develop a combined SIT/IIT protocol which could potentially be used for the population suppression of *D. suzukii*. The two *Wolbachia* candidate strains (*w*Ha and *w*Tei) suggested by Cattel et al. (2018) were used and three low irradiation doses were evaluated. The effect of the *Wolbachia* infection and irradiation on the adult emergence, longevity and flight ability were also assessed.

## Materials and methods

### *D. suzukii* lines and rearing conditions

Four lines harboring the same genetic background from France, but with different infection status were obtained in a previous study by Cattel and colleagues (2018) through microinjections of *Wolbachia* strains from other *Drosophila* species into *D.suzukii*; a *Wolbachia*-free line (un-Fr), a *wSuz*-infected line (*wSuz*-Fr) and two trans-infected lines (*w*Ha-Fr and *w*Tei-Fr). These two exogenous *Wolbachia* strains, *w*Ha and *w*Tei, were shown to induce high CI levels in *D. suzukii* trans-infected lines despite the presence of the natural *Wolbachia* infection *w*Suz (Cattel et al. 2018). All these lines were kindly provided by the Laboratory of Biometry and Evolutionary Biology, University Lyon 1, France.

A naturally infected *w*Suz line (hereinafter mentioned as “*w*Suz-IPCL”) was also obtained from the Agricultural Entomology Unit of the Edmund Mach Foundation in San Michele All’Adige, Trento Province, Italy and maintained in laboratory conditions at the IPCL for 55 generations before its use in this study. A *Wolbachia*-free line (hereinafter mentioned as “uninfected”) was obtained after treatment of the *w*Suz-IPCL line with 0.25 mg ml^−1^ tetracycline added in the diet as used in Cattel et al. 2016. The tetracycline treatment lasted for four consecutive generations. In the next two generations, the flies were reared in a diet “inoculated” with *w*Suz male feces to restore their gut-associated microbial community. After their gut microbiota recovery, the flies were reared in normal standard diet. The absence of *Wolbachia* was confirmed by PCR targeting the WSpec 16S rDNA region (Werren and Windsor 2000). The WspecF 5′-CAT ACC TAT TCG AAG GGA TAG-3′ and WspecR 5′-AGC TTC GAG TGA AAC CAA TTC-3′ primers used for the PCR reaction amplify an approximately 440-bp fragment. The amplification reaction mixture contained 1X Taq PCR Master Mix kit (QIAGEN, Cat No./ID: 201445), which is a premixed solution consisting of Taq DNA Polymerase, PCR Buffer and dNTPs. In addition, 1 μM of each primer, 1μL of DNA template and deionized sterile water to a final volume of 25 μl were added. The PCR cycle conditions included an initial step at 94 °C for 3 min, followed by 35 cycles of 94 °C for 45 s, 55 °C for 45 s, 72 °C for 1 min, and a final elongation step at 72 °C for 10 min.

All *D. suzukii* lines were reared in a carrot-based diet containing: 1% agar, 3.75% sugar, 3.75% carrot powder (Kanegrade Ltd), 1.5% yellow corn meal, 2.25% inactive dry yeast and 0.5% propionic acid, and maintained at a room with 23–24 °C temperature, 45–50% humidity and natural light conditions.

### Cytoplasm introgression

Introgression experiments were performed to align all *Wolbachia* strains under a common genetic background. Females from *w*Ha-Fr, *w*Tei-Fr and *w*Suz-Fr lines were crossed with males from the *w*Suz-IPCL line. The female offspring of these crosses were then backcrossed to *w*Suz-IPCL males for a total of eight generations. After the final backcrossing, the three lines, hereinafter mentioned as “*w*Ha” “*w*Tei” and “*w*Suz”, were checked with PCR to confirm *Wolbachia* presence, as described above. Multi-Locus Sequence Typing (MLST) analysis of *Wolbachia* was performed by PCRs targeting six genes (*wsp*, *gatB*, *coxA*, *hcpA*, *ftsZ*, *fbpA*) as described in Baldo et al. (2006) in order to verify that the *Wolbachia* strain was the one expected in the lines. PCR products were sequenced, and sequences were aligned using the ClustalW algorithm in the BioEdit v.7.0.5 software (Hall 1999).

### Life-history traits of *D. suzukii* lines

Unless otherwise stated, in all experiments freshly emerged adults were sexed and placed separately in vials with standard diet until aged 5–6 days old. At that time point, the adults were used for the assays described below. All experiments were performed at constant laboratory conditions as described above.

To estimate the effect of *Wolbachia* on the fecundity and hatch rate of the lines, we placed 10 virgin males and 10 virgin females in a standard diet vial and allowed them to mate for 48 h. Males were then removed, and females laid eggs individually in Petri dishes containing a substrate of raspberry-juice agar. The females were transferred daily to a fresh substrate until three changes had been completed (72 h of egg laying in total). The number of eggs was counted daily, and the hatching was recorded 48 h after egg laying to ensure that all eggs were given enough time to hatch. Three replicates were performed for each of the four lines. Fecundity was calculated as the average number of eggs laid per female, and hatch rate was determined as the number of hatched eggs per the total number of eggs laid.

Subsequently, the larvae were placed in a Petri dish with standard diet and allowed to pupate. The pupal weight was measured 1 day before adult emergence. Pupae from each line were sorted into 10 replicate groups with 5 pupae in each group and they were weighted.

To check whether the progeny balance is affected by *Wolbachia*, we placed 10 virgin males and 10 virgin females in a standard diet vial and allowed them to mate for 48 h. Males were removed, and females laid eggs individually in vials with standard diet for 48 h. Females were then transferred to fresh vials and allowed again to lay eggs for another 48 h. Adult emergence was recorded daily, and sex ratio was determined as proportion of males per total number of adults. Three replicates were performed per line.

Adult longevity was assessed by placing 15 newly emerged males and females separately in vials with standard diet. Mortality was monitored daily and until all adults were dead. The experiment was performed in triplicates per line per sex.

### Cytoplasmic incompatibility (CI) assays

The CI expression levels of *w*Ha and *w*Tei males were checked in crosses with either *w*Suz or uninfected females. All CI crosses were single, and we used 2-to-3-day-old virgin males and 5-to-6-day-old virgin females. The couple was placed in a vial with a fresh raspberry and allowed to mate for 24 h. The vials were inspected multiple times during the day, and the couples that mated for at least 15 min were recorded and used downstream. The couples for which no mating was observed were discarded. Males were then removed, and females were allowed to lay eggs individually in a raspberry-juice agar substrate for 48 h. After that, females were transferred again individually in a new Petri dish for another 48 h. Only females that laid at least 20 eggs in total were included in the analysis. At least 19 repetitions were performed for each cross type (compatible and incompatible).

The corrected index of CI (CI_corr_) (Poinsot et al. 1998) was used to minimize the variation effect that the natural embryonic mortality could have on the CI level estimation. This mortality is not related to CI, and it is defined by the compatible cross scheme. CI_corr_ is calculated as: CI_corr_ = [(CI_obs_ − CCM)/(100 − CCM)] × 100, where CI_obs_ is the percentage of unhatched eggs in the incompatible cross, and CCM is the mean mortality observed in the control crosses.

### Effect of male age on CI levels

Male age is a factor known to potentially reduce the CI levels. We investigated the impact of male age on the CI intensity by crossing *w*Ha and *w*Tei males with *w*Suz or uninfected females. Males and females were sexed and placed separately in vials with standard diet. The males that were used in the crosses were 2–3, 5–6 or 9–10 days old, while in all cases the females were 5–6 days old. Mass crosses of ten males and ten females were performed and allowed to mate for 48 h. Males were removed, and females were placed in a common cage to lay eggs in raspberry-juice agar for 48 h. Control crosses of the uninfected and *w*Suz lines were used to quantify the effect of male age in the absence of CI-inducing *Wolbachia* strains. The CI corrected index (CI_corr_) was used again for the assessment of the male age effect. Six replicates were performed for each cross type.

### Effect of irradiation dose on egg hatch rate

A ^60^Co irradiator (Gamma Cell-220, Nordion, Canada) was used for the irradiation of *w*Ha and *w*Tei pupae. The pupae of each strain were collected one day before emergence, placed in a 60 × 15 mm Petri dish and irradiated. Based on previous knowledge about the irradiation dose required for sterilizing completely *D. suzukii* females (Krüger et al. 2018; Lanouette et al. 2017; Sassù et al. 2019), the doses tested here were 45, 60 and 90 Gy. After irradiation, the pupae were placed in a cage and the emerged adults were collected next day. The collection of irradiated adults lasted for 24 h after irradiation and all adults that emerged later were discarded, as they were considered young at the time of irradiation. Irradiated males and females were sexed and placed separately in vials with standard diet for 5–6 days. Virgin fertile *w*Suz males and females were also collected simultaneously. To assess the egg hatch rate of the *w*Ha and *w*Tei adults, fifteen irradiated males and females from each strain were crossed with fifteen fertile *w*Suz females and males, respectively, and allowed to mate and oviposit in raspberry-juice agar substrate for 24 h. The oviposition substrate was replaced daily for three consecutive days. All control crosses of the fertile *w*Suz, *w*Ha and *w*Tei adults were also performed. The eggs were counted and transferred in Petri dishes with standard diet to ensure that all nutrients required for the larval development were supplied. Egg hatching was recorded 48 h after oviposition. The experiment was performed in two different time points. At first the doses 60 and 90 Gy were applied, and three replicates per cross type, per dose were performed. At the next generation, the doses 45 and 60 Gy were applied and three replicates per cross type, per dose were performed. The 60 Gy irradiation dose was performed twice as a reference to normalize any effect of time in our data.

Three pieces of Gafchromic^®^HD-V2 dosimetry films (International Specialty Products, NJ, USA) (10 × 10 mm) were centered on top of the Petri dish before the irradiation. Twenty-four hours after irradiation, the films were read by a Radiochromic reader (FWT-92D, Far West Technology, Inc., Goleta, CA, USA) to confirm the irradiation dose that the pupae had received. Dosimetry was performed according to the manual and all readings were within the 95% confidence intervals (Gafchromic ^®^ Dosimetry System for SIT. Standard Operating Procedure 2004).

### Effect of irradiation on adult emergence rate

*w*Ha and *w*Tei pupae were irradiated at 0 and 45 Gy as described above. The pupae were clustered in groups of fifteen, placed in vials and left to emerge. Non-irradiated *w*Suz pupae were also included as control. The number of emerged adults and their sex were recorded in all cases. Three replicates were performed for each line.

### Effect of irradiation on adult longevity

The longevity of irradiated and non-irradiated adults was assessed both for males and females. *w*Ha and *w*Tei pupae were irradiated at 0 and 45 Gy, and freshly emerged adults were sexed and placed in vials containing an agar-sugar substrate (1% agar, 10% sugar, 1% yeast dissolved in water). Fifteen adults were included per vial, and three replicates per line, treatment, and sex were performed.

### Effect of irradiation on flight ability

Irradiated at 45 Gy and non-irradiated *w*Ha and *w*Tei pupae were used to assess the effect of irradiation on the adult flight ability. *w*Suz non-irradiated pupae were also included as control. After irradiation, pupae were placed at the bottom of an open Petri dish. A black plexiglass tube was adjusted over the Petri dish and the tube’s internal site was coated with unscented talcum powder to prevent flies from crawling out of the tube (FAO/IAEA/USDA 2014). Flies were periodically aspirated from the vicinity of the tubes to avoid falling-back into the tubes. Five replicates with 15 pupae each were set up per treatment.

### Statistical analysis

All data were examined for normality using the Shapiro–Wilk normality test. In data sets where the normality assumption was violated, nonparametric tests were applied. Analysis of variance (ANOVA) was used to examine the significance of interactions between factors. Interactions that were not significant were excluded and models were simplified. *Lsmeans* (Lenth 2016) and *multcomp* (Hothorn et al. 2008) packages were used for the pairwise comparisons of the fitted model estimates. In all datasets, the *Wolbachia* strain was included as a fixed factor and replicates as a random factor. Pupal weight data were analyzed using a linear mixed-effect model and they were square transformed to improve normality of the residual errors. A GLMM (binomial family) was used for the analysis of the CI, age of males’ effect, emergence rate, flight ability, and hatching rates. Fecundity and sex ratio data were analyzed with a GLMM (Poisson family). The survivorship curves were calculated using a Kaplan–Meier approach (*survfit* package) (Kaplan and Meier 1958). All statistical analyses were performed using R version 3.5.2 (R Core Team 2018). The package *lme4* was used for all mixed models (Bates et al. 2015). The package *survival* was used for modeling the longevity data (Therneau 2015). In all cases the mean ± standard error is reported. The statistical results for all datasets are available in Online Resource 4.

## Results

### Life-history traits of the introgressed *D. suzukii* lines

We did not detect any effect of the infection status on the fecundity of the lines after 72 h of egg laying. Despite the fact that the mean egg production of the uninfected line was considerably higher (42.7 ± 4.58) compared to the *w*Ha (22.7 ± 2.86), *w*Tei (22 ± 2.9) and *w*Suz (26 ± 3.32) lines (Online Resource 1a), the differences among the lines were not significant (uninfected-*w*Ha GLMM: *z* = − 1.608, *p* = 0.374; uninfected-*w*Tei GLMM: *z* = − 1.833, *p* = 0.258; uninfected-*w*Suz GLMM: *z* = − 1.093, *p* = 0.694). Absence of any effect was also observed on the hatch rates of the lines (Online Resource 1b), although the uninfected line showed marginally higher hatch rate (91.5% ± 1.67), compared to the *w*Ha, *w*Suz and *w*Tei lines (86% ± 5.91, 84.5% ± 6.21 and 77.9% ± 7.48, respectively).

The pupal weight data showed that the uninfected and the *w*Suz lines had higher average weight (17.2 ± 0.24 mg and 16.7 ± 0.43 mg, respectively) compared to the *w*Ha and *w*Tei lines (14.7 ± 0.52 mg and 15.5 ± 0.39 mg, respectively) (Online Resource 2). The data showed that the infection status was related to the pupal weight (*F*_*3,36*_= 7.509, *p* = 0.0005).

The infection status did not result in any sex ratio imbalances in the four *D. suzukii* lines. No significant differences were observed among the lines (Kruskal–Wallis; *χ*^2^ = 6.9361, *df* = 3, *p* = 0.073). The sex ratio in the uninfected line was 0.487 ± 0.03, in the *w*Ha line 0.506 ± 0.03, in the *w*Suz line 0.557 ± 0.02 and in the *w*Tei line 0.498 ± 0.03.

The presence of *Wolbachia* did not appear to impact the adult longevity. Due to the continuous supply of an energy source and water, the mortality rates were extremely low during the first 40 days of the experiment. Therefore, the mortality was recorded daily up to 58 days and after that, the experiment was discontinued, since the evaluation of such long longevity times is untenable from an application point of view. Both females (log-rank test; *χ*^*2*^= 3.1, *df* = 3, *p* = 0.4) and males (log-rank test; *χ*^*2*^= 5.2, *df* = 3, *p* = 0.2) presented the same survival probability, regardless of the infection status (Online Resource 3a, b).

### CI expression levels

**Fig. 1:**
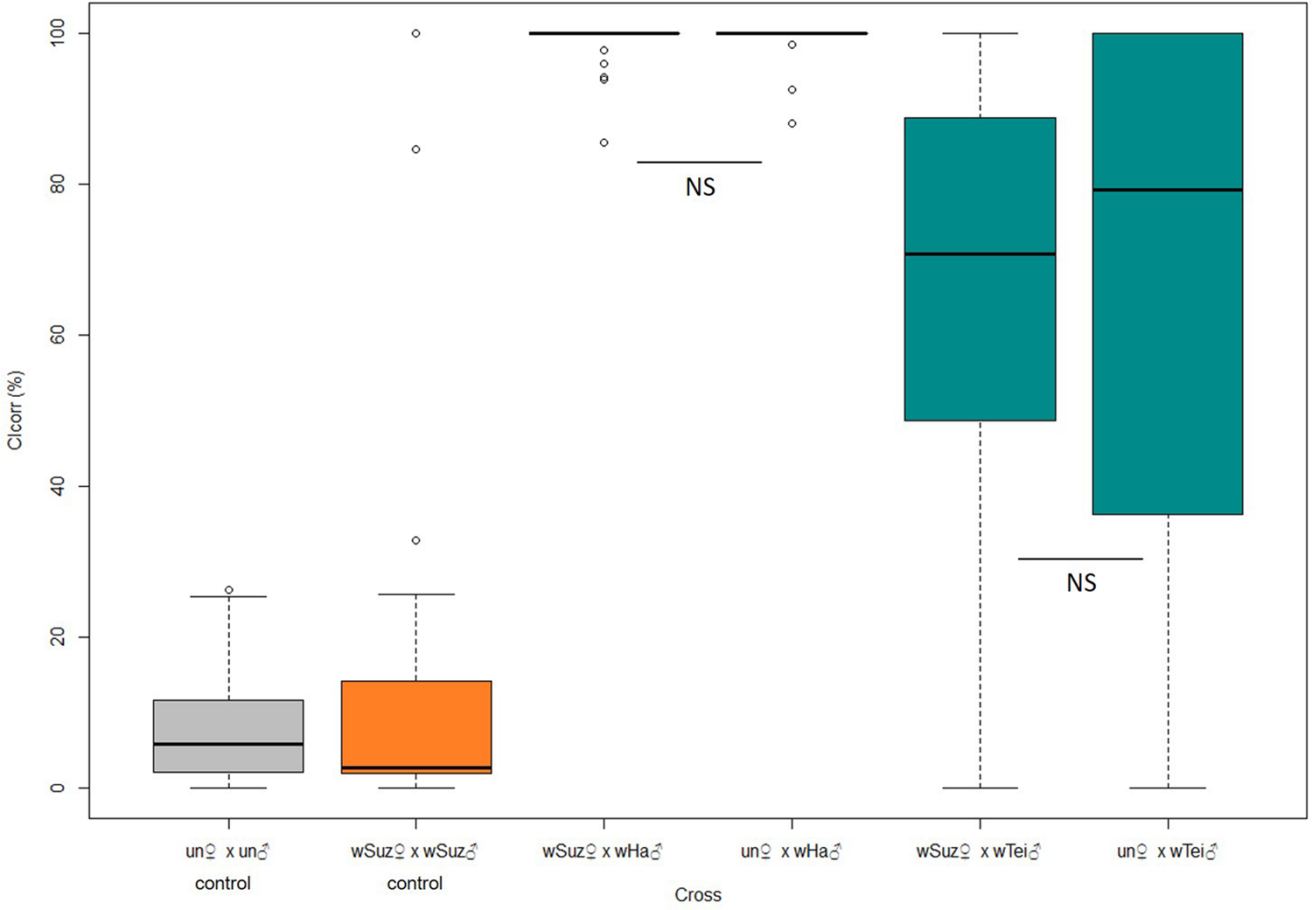
Cytoplasmic incompatibility levels estimated in individual crosses in *D. suzukii* lines. In all crosses the males were 2–3 days old and the females were 5–6 days old. The corrected index of CI (CI_corr_) was used to account for the basal embryonic mortality and estimate only the CI-related mortality. A GLMM (binomial family) analysis was performed to determine the differences between the crosses. (un♀ × un♂: N = 24; *w*Suz♀ × *w*Suz♂: N = 20; *w*Suz♀ × *w*Ha♂: *N* = 22; un♀ × *w*Ha♂: N = 20; *w*Suz♀ × *w*Tei♂: N = 26; un♀ × *w*Tei♂: *N* = 19; Confidence level used: 0.95, alpha = 0.05)

We determined the CI levels in single-pair crosses that included males infected either with *w*Ha or *w*Tei and females uninfected or infected with *w*Suz (Fig. 1 ). Our results showed that *w*Ha induces strong CI when crossed with uninfected or *w*Suz-infected females (98.9% ± 0.68 and 98.5% ± 0.74, CI_corr_, respectively). The difference between uninfected and *w*Suz-infected females was not statistically significant (GLMM: *z* = − 0.305, *p* = 1) indicating that CI induced by *w*Ha is not rescued by the natural *w*Suz infection. On the other hand, crosses with *w*Tei males presented significantly lower CI levels compared to the respective ones with *w*Ha (un ♀ × *w*Tei ♂ - un ♀ × *w*Ha ♂ GLMM: *z* = -6.014, *p* = < 1e-05; *w*Suz ♀ × *w*Tei ♂ - *w*Suz ♀ × *w*Ha ♂ GLMM: *z* = 6.829, *p* = < 1e − 05). The CI_corr_ values were 64.8% ± 8.37 for crosses with uninfected females and 67.4% ± 5.6 for crosses with *w*Suz-infected females.

### Effect of male age on CI levels

**Fig. 2:**
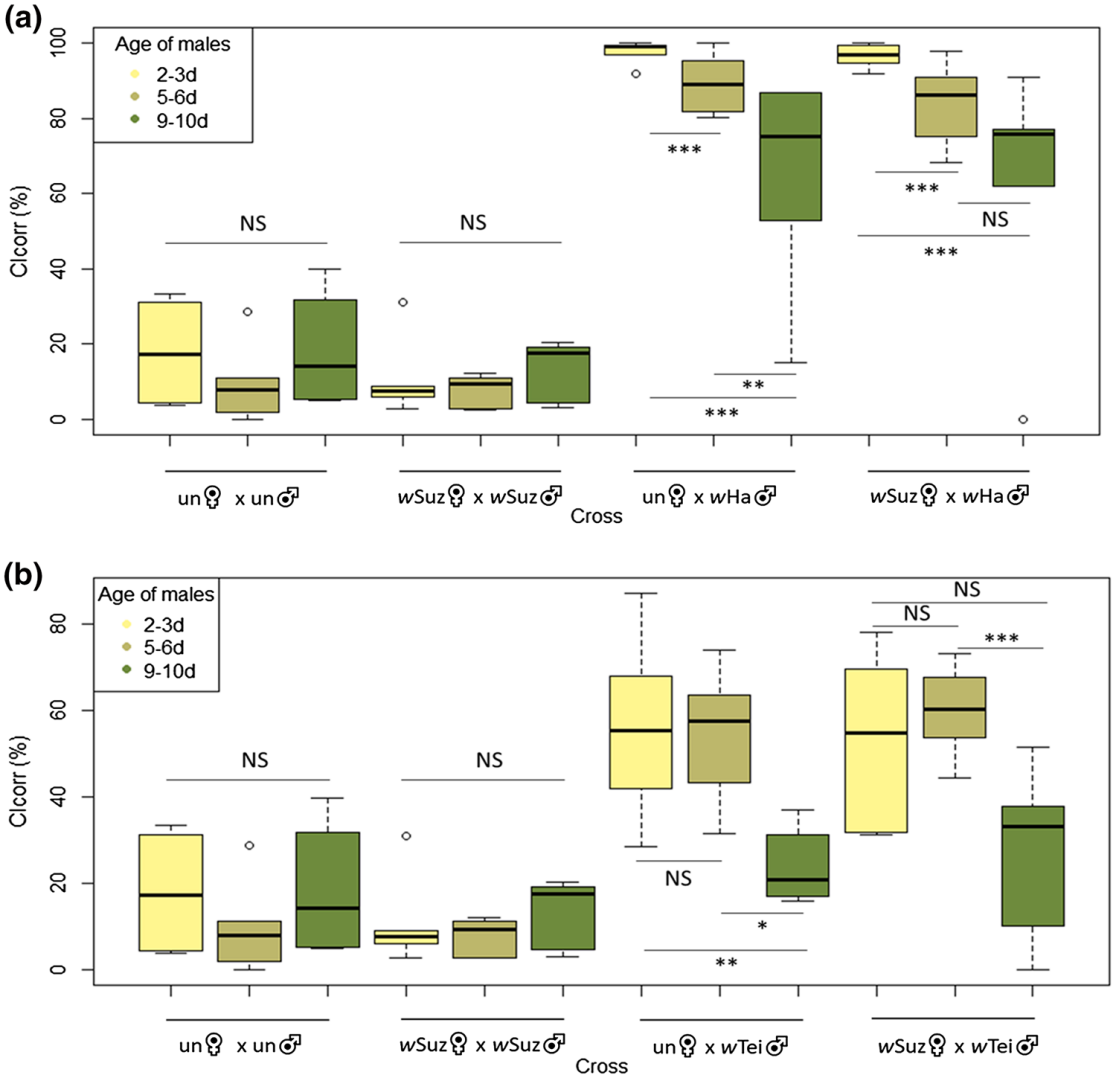
Effect of male age on CI levels in *D. suzukii* crosses. In all crosses the females were 5–6 days old. **a** Crosses with *w*Ha infected males; **b** crosses with *w*Tei infected males. The corrected index of CI (CI_corr_) was used to account for the basal embryonic mortality and estimate only the CI-related mortality. A GLMM (binomial family) analysis was performed to determine the differences between the crosses. ***p < .001, **p < .01, *p < .05 (Confidence level used: 0.95, alpha = 0.05; *N* = 60)

In our control crosses, there was no significant difference among the different age groups of the males. We observed significant differences in the CI_corr_ levels in crosses with *w*Ha males that were dropping as the age of the males was increased (Fig. 2a). In crosses between uninfected females and *w*Ha infected males, the CI_corr_ was 97.7% ± 1.25 in males aged 2–3 days old and dropped to 65.3% ± 11.26 in males aged 9–10 days old (Fig. 2a). A notable decrease was also observed in crosses with *w*Suz-infected females (96.6% ± 1.31 CI_corr_ in 2-to-3-day-old *w*Ha males and 63.5% ± 13.2 CI_corr_ in 9-to-10-day-old *w*Ha males) thus indicating an effect of the male age on the CI intensity of the *w*Ha line. The age of males affected the CI intensity also in crosses with *w*Tei males. CI_corr_ dropped from 56% ± 8.28 (2 to 3 days old) to 23.8% ± 3.42 (9 to 10 days old) and from 53.3% ± 7.87 (2 to 3 days old) to 27.6% ± 7.71 (9 to 10 days old) in crosses with uninfected and *w*Suz-infected females, respectively (Fig. 2b).

### Effect of irradiation dose on egg hatch rate

**Fig. 3:**
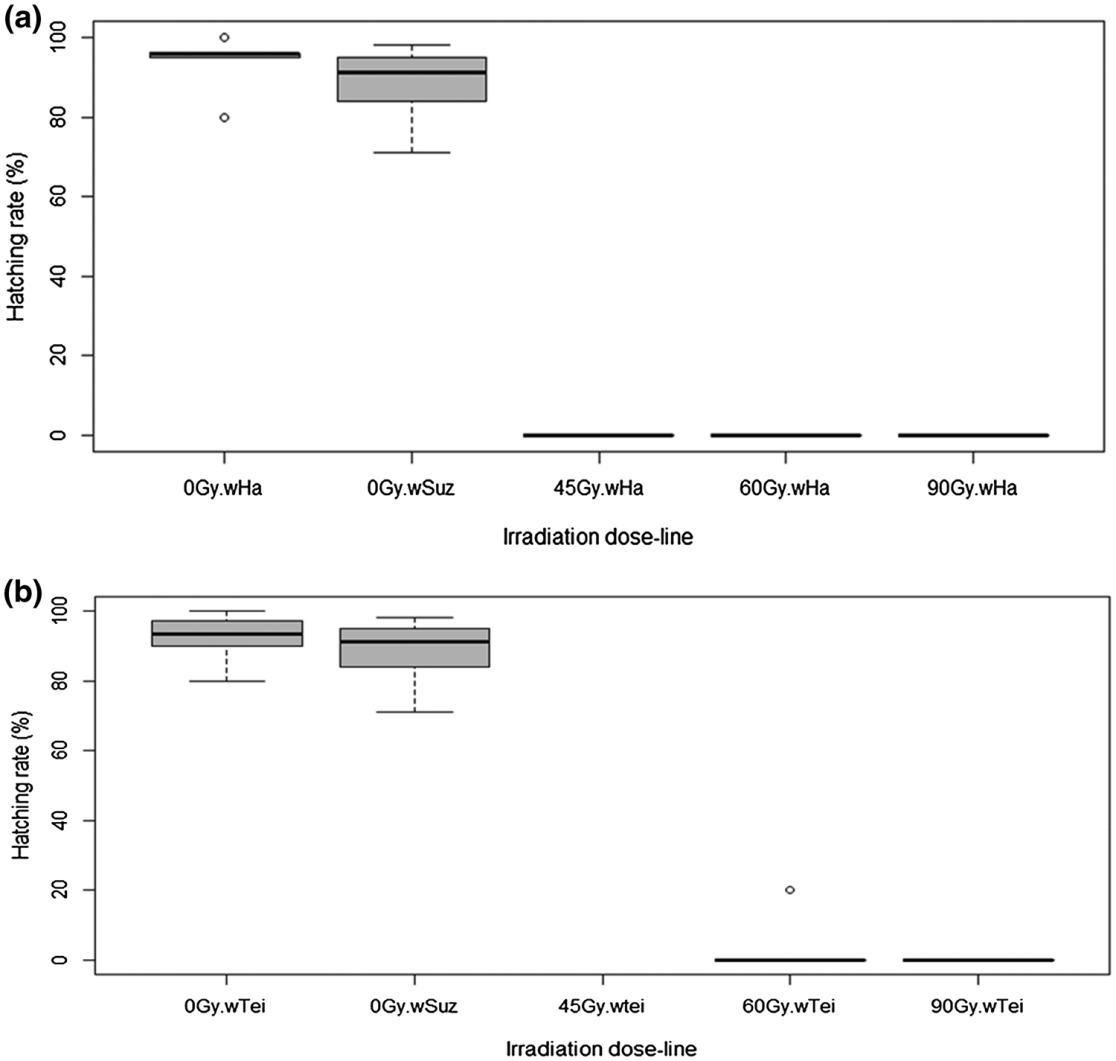
Hatching rates of crosses between irradiated, *Wolbachia*-infected females and fertile, *w*Suz males. **a** Crosses with *w*Ha infected females; (Kruskal–Wallis Chi-squared = 20.334, *df* = 4, *p* value = 0.0004289); [0 Gy.*w*Ha: *w*Ha ♀ × *w*Ha ♂ (fertile, non-irradiated females and males), 0 Gy.*w*Suz: *w*Suz ♀ × *w*Suz ♂ (fertile, non-irradiated females and males), xxGy.*w*Ha: *w*Ha ♀ × *w*Suz ♂ (irradiated *w*Ha females crossed with fertile *w*Suz males)], **b** Crosses with *w*Tei infected females; (Kruskal–Wallis Chi-squared = 19.338, *df* = 4, *p* value = 0.0006743); [0 Gy.*w*Tei: *w*Tei ♀ × *w*Tei ♂ (fertile, non-irradiated females and males), 0 Gy.*w*Suz: *w*Suz ♀ × *w*Suz ♂ (fertile, non-irradiated females and males), xxGy. *w*Tei: *w*Tei ♀ × *w*Suz ♂ (irradiated *w*Tei females crossed with fertile *w*Suz males)]. Pupae were irradiated at 45, 60 and 90 Gy. All adults used for the crosses were 5–6 days old. A GLMM (binomial family) analysis was performed to determine the differences between the crosses. alpha = 0.05

**Fig. 4:**
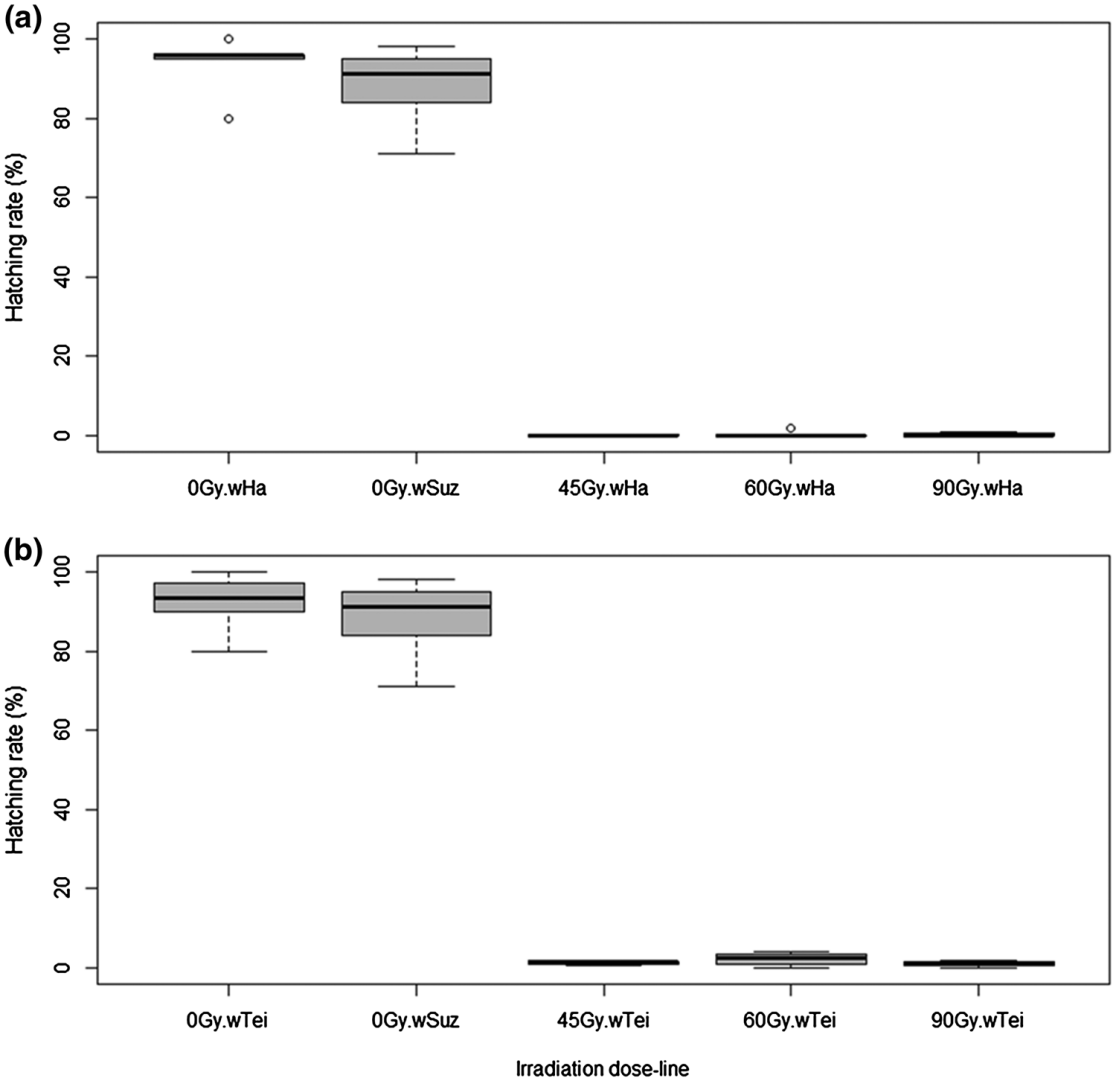
Hatching rates of crosses between irradiated, *Wolbachia*-infected males and fertile, *w*Suz females. **a** Crosses with *wHa* infected males; (Kruskal–Wallis Chi-squared = 18.814, *df* = 4, *p* value = 0.0008548); [0 Gy.*w*Ha: *w*Ha ♀ × *w*Ha ♂ (fertile, non-irradiated females and males), 0 Gy.*w*Suz: *w*Suz ♀ × *w*Suz ♂ (fertile, non-irradiated females and males), xxGy.*w*Ha: *w*Suz ♀ × *w*Ha ♂ (fertile *w*Suz females crossed with irradiated *w*Ha males)], **b** Crosses with *w*Tei infected males; (Kruskal–Wallis Chi-squared = 17.995, *df* = 4, *p* value = 0.001237); [0 Gy.*w*Tei: *w*Tei ♀ × *w*Tei ♂ (fertile, non-irradiated females and males), 0 Gy.*w*Suz: *w*Suz ♀ × *w*Suz ♂ (fertile, non-irradiated females and males), xxGy. *w*Tei: *w*Suz ♀ × *w*Tei ♂ (fertile *w*Suz females crossed with irradiated *w*Tei males)]. Pupae were irradiated at 45, 60 and 90 Gy. All adults used for the crosses were 5–6 days old. A GLMM (binomial family) analysis was performed to determine the differences between the crosses. alpha = 0.05

Egg hatch rate data revealed an effect of radiation on the progeny production in the *Wolbachia*-infected lines (Table 1). In the control crosses of the *w*Ha, *w*Tei and *w*Suz lines (which consisted of non-irradiated, fertile females and males of each line), the hatching rate was 93.8% ± 2.85, 92.3% ± 2.97, and 88.4% ± 3.98, respectively. When irradiated *w*Ha females were crossed with fertile *w*Suz males, no eggs were laid at 60 and 90 Gy, while we collected only 6 eggs at 45 Gy which did not hatch (Fig. 3a). The irradiated *w*Tei females laid 1 egg at 45 Gy that did not hatch, 6 eggs at 60 Gy out of which only one hatched, but the larva died before pupation and 1 non-hatched egg at 90 Gy (Fig. 3b). Significant decrease in egg hatch rate was also observed in the crosses between irradiated males and fertile *w*Suz females. When *w*Ha males were irradiated at 45 Gy and crossed with fertile *w*Suz females, only 1 egg hatched out of the 782 collected eggs (0.13%). At 60 Gy we collected 926 eggs and only 2 of them hatched (0.23%) and at 90 Gy the egg hatching was 0.33% (1 hatched out of the 300 collected eggs) (Fig. 4a). In all the above cases involving irradiated *w*Ha males, the larvae died before the pupation stage (Table 1). In the case of *w*Tei irradiated males, at 45 Gy the egg hatching was 1.1% (17 out of 1537 eggs hatched), at 60 Gy it was 2.4% (25 out of 1048 eggs hatched) and at 90 Gy it was 1% (3 out of 300 eggs hatched) (Fig. 4b). The number of F1 progeny (pupae and emerged adults) coming from crosses with irradiated *w*Tei males is described in Table 1.

**Table 1:** Effect of irradiation on the egg hatching and adult emergence of the *w*Ha and *w*Tei *D. suzukii* lines, when females or males of these lines are irradiated

Cross	Irradiation dose (Gy)	Number of eggs	Number of hatched eggs	Number of pupae	Emerged females	Emerged males
*w*Ha ♀ × *w*Ha ♂	0	600	563	453	198	190
*w*Tei ♀ × *w*Tei ♂	0	600	554	418	200	192
*w*Suz ♀ × *w*Suz ♂	0	593	524	435	187	209
*w*Ha ♀ × *w*Suz♂	45	6	0	0	0	0
*w*Ha ♀ × *w*Suz♂	60	0	0	0	0	0
*w*Ha ♀ × *w*Suz♂	90	0	0	0	0	0
*w*Tei ♀ × *w*Suz♂	45	1	0	0	0	0
*w*Tei ♀ × *w*Suz♂	60	6	1	0	0	0
*w*Tei ♀ × *w*Suz♂	90	1	0	0	0	0
*w*Suz ♀ × *w*Ha♂	45	782	1	0	0	0
*w*Suz ♀ × *w*Ha♂	60	926	2	0	0	0
*w*Suz ♀ × *w*Ha♂	90	300	1	0	0	0
*w*Suz ♀ × *w*Tei♂	45	1537	17	16	9	5
*w*Suz ♀ × *w*Tei♂	60	1048	25	19	7	11
*w*Suz ♀ × *w*Tei♂	90	300	3	1	0	1

### Effect of irradiation dose on adult emergence, longevity and flight ability

**Fig. 5:**
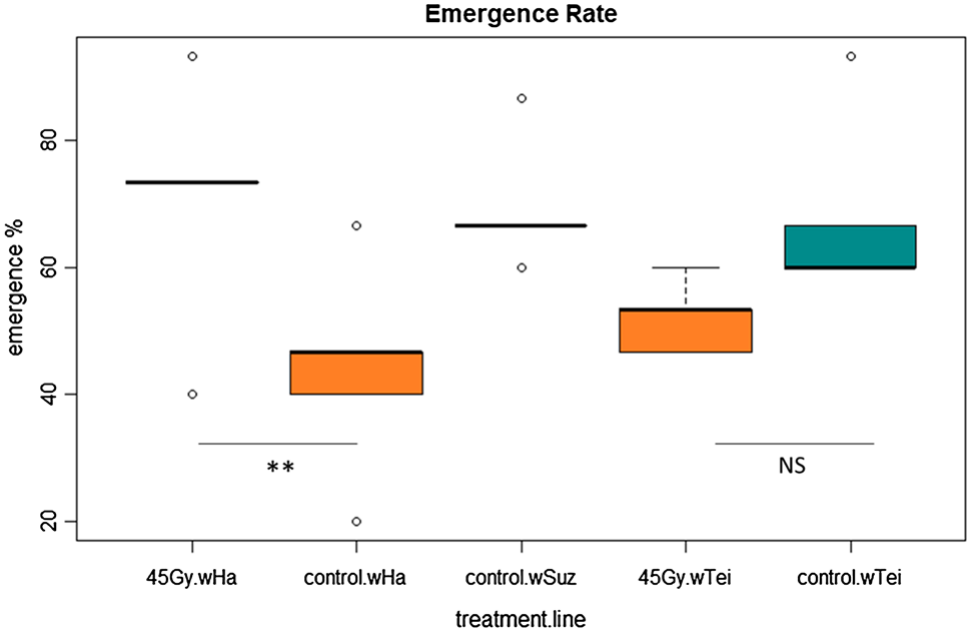
Effect of 45 Gy irradiation dose on the emergence rate of irradiated and non-irradiated pupae. A GLMM (binomial family) analysis was performed to determine the differences between the groups. **p < .01 (Kruskal–Wallis Chi-squared = 11.21, *df* = 4, *p* value = 0.0243, *N* = 75)

The effect of irradiation at 45 Gy on adult emergence was assessed both for *w*Ha and *w*Tei adults. The emergence rate was significantly influenced by the irradiation dose (Kruskal–Wallis; *χ*^2^ = 11.21, *df* = 4, *p* = 0.0243). We observed significant differences between *w*Ha adults irradiated at 45 Gy and *w*Ha non-irradiated adults (GLMM: *z* = − 3.256, *p* = 0.0099), while the differences between irradiated and non-irradiated *w*Tei adults were not significant (GLMM: *z* = 1.989, *p* = 0.2709) (Fig. 5).

**Fig. 6:**
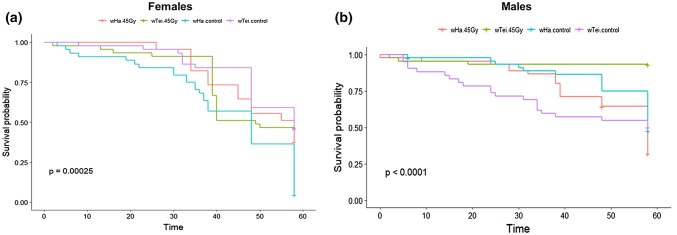
Effect of 45 Gy irradiation dose on **a** female and **b** male longevity. Flies were provided with an agar-sugar substrate and dead flies were recorded daily. Significant differences were measured with a log-rank test. The *x*-axis represents time in days

Adult longevity data showed a significant effect of irradiation on the survival days of both females and males (♀log-rank test: *χ*^*2*^= 19.2, *df* = 3, *p* = 2e−04; ♂ log-rank test: *χ*^*2*^= 27.1, *df* = 3, *p* = 5e−06) (Fig. 6a). Irradiated *w*Ha females had significantly shorter longevity times compared to the non-irradiated ones (♀*w*Ha irradiated vs. *w*Ha non-irradiated: *χ*^*2*^= 9.3, *df* = 1, *p* = 0.002), while the same was not true for *w*Tei females (♀*w*Tei irradiated vs. *w*Tei non-irradiated: *χ*^*2*^= 0.3, *df* = 1, *p* = 0.6). The opposite pattern was observed for the males; the difference was non-significant for the *w*Ha males (♂*w*Ha irradiated vs. *w*Ha non-irradiated: *χ*^*2*^= 2.2, *df* = 1, *p* = 0.1), but significant difference was observed for the *w*Tei males (♂*w*Tei irradiated vs. *w*Tei non-irradiated: *χ*^*2*^= 18.8, *df* = 1, *p*  = 1e − 05) (Fig. 6b).

**Fig. 7:**
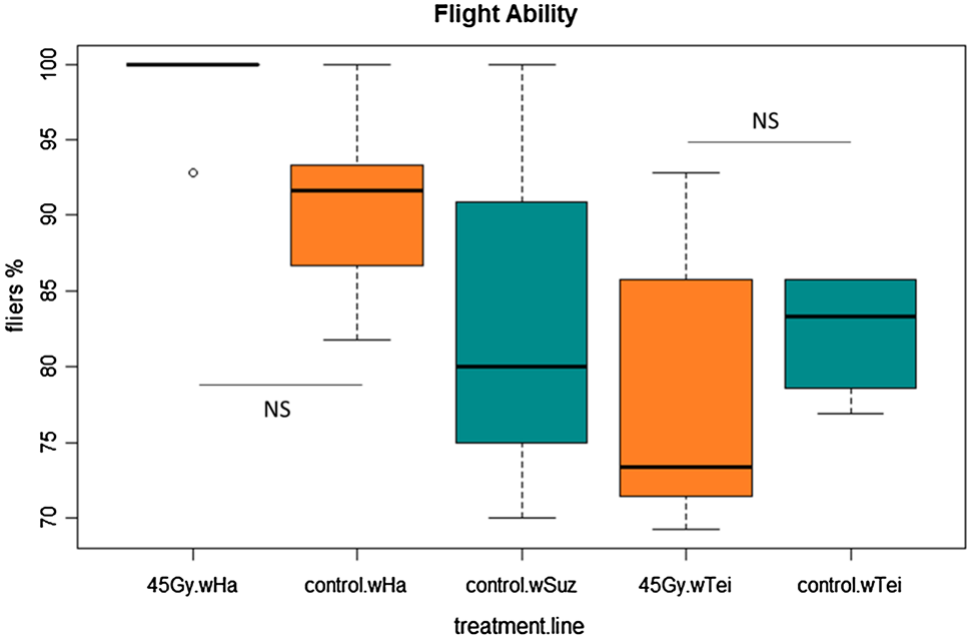
Effect of 45 Gy irradiation dose on the flight ability of irradiated and non-irradiated adults. A GLMM (binomial family) analysis was performed to determine the differences between the groups

The flight ability data showed that there was no significant difference between the irradiated and non-irradiated flies. In the *w*Ha line, the fliers coming from the irradiated pupae were 98.6% ± 1.42 and 90.7% ± 3.07 from the untreated pupae (GLMM: *z* = −1.750, *p* = 0.3825). The same result was observed for the *w*Tei individuals, where the irradiated fliers were 78.5% ± 4.6 and the control *w*Tei fliers were 82.1% ± 1.83 (GLMM: *z* = 0.517, *p* = 0.9844) (Fig. 7).

## Discussion

Our main goal in this study was to develop a combined SIT and IIT approach which could potentially be used for the control of *D. suzukii* populations. A previous study determined the optimum irradiation dose for an adequate *D. suzukii* male sterility level (99.67%) at 200 Gy (Krüger et al. 2018). In addition, Lanouette and her colleagues (2017) showed that 96% of sterility can be achieved if males are irradiated at 120 Gy, while full female sterility was achieved at 75 Gy in both studies. Assessing the effect of high irradiation doses on several quality parameters (emergence rate, flight ability, longevity and sex ratio) did not indicate any alterations or decay in performance in both studies (Krüger et al. 2018; Lanouette et al. 2017).

Given the above promising results and considering the urge to develop a sustainable and environmentally sound approach, we aimed to establish a combined SIT/IIT protocol that would require lower irradiation doses that may not affect the quality of sterile males, as shown for the mosquito vector species *Ae. albopictus* (Zhang et al. 2015b; Zheng et al. 2019) and would act as a complementary tool for *D. suzukii* management. Based on the female sterility dose suggested by Krüger et al. (2018) and Lanouette et al. (2017), we tested the irradiation doses at 45, 60 and 90 Gy on *wHa* and *wTei* trans-infected individuals and observed complete sterility both for *w*Ha and *w*Tei females at all three doses. Similar encouraging results were also obtained for males (99.887% sterility for *w*Ha males and 98.9% sterility for *w*Tei males at 45 Gy). None of the quality control parameters tested at 45 Gy was negatively affected, excluding the irradiated wHa adults that were positively affected showing elevated emergence rate compared to the non-irradiated ones.

The noteworthy difference in the irradiation dose between *w*Suz-infected males, as defined by previous studies (Krüger et al. 2018; Lanouette et al. 2017), and *w*Ha and *w*Tei infected males, as defined by the present study, demonstrates an engagement of the *Wolbachia* infection in the effect prompted by irradiation on the egg hatching rate. Irradiation is known to induce the formation of free radicals that create dominant lethal mutations in the germ cells (Bakri et al. 2005). The low irradiation dose we determined for the complete sterility of the *w*Ha and *w*Tei lines might suggest a higher susceptibility of the trans-infected lines to the oxidative stress caused by irradiation (Monnin et al. 2016). Future research is required to dissect the mechanism underlying the antioxidant capacity of the two trans-infected lines. The increased emergence rate observed for the irradiated *w*Ha adults could be attributed to the hormesis hypothesis. Hormesis has been described as the stimulatory outcome observed after mild or sublethal stress levels and it has long been realized in insects (Le Bourg 2010; Cutler 2013). Low stress levels can have beneficial effects on several biological traits of insects, and this could explain why we noticed a performance gain in terms of adult emergence in the 45 Gy-irradiated *w*Ha line and not in the control one. The increased adult emergence after a low irradiation dose for the *w*Ha line is an add-in value for the combined SIT/IIT approach for *D. suzukii*.

The presence or absence of *Wolbachia*, as well as the different *Wolbachia* strains and the host genome can lead in phenotypic variations across host species. Martinez and colleagues (2017) showed that the antiviral protection phenotype exerted by the same symbiont was mostly dependent on the *Wolbachia* strain rather than the host species. On the other hand, the host nuclear background is actively involved in the expression of fitness costs or benefits within the same species and it can be a leading factor in delivering decisive *Wolbachia* phenotypes (Dean 2006; Mouton et al. 2007; Poinsot et al. 1998; Veneti et al. 2012). In this study, we assessed the biological traits of two *Wolbachia*-infected *D. suzukii* lines which are maintained under a different genetic background than the one they were developed in. Our results clearly demonstrated that fecundity, hatch rate, sex ratio and adult longevity are not affected by the infection status. Mazzetto et al. (2015) have previously reported a beneficial effect of *w*Suz infection on female fecundity compared to antibiotic-treated individuals which was not confirmed by our study. On the contrary, we observed a negative impact of the *w*Ha and *w*Tei infections on the pupal weight. The pupal weight can be used as a proxy to estimate the adult size (Nash and Chapman 2014). Low pupal weight might indicate weak adults with decreased flight ability (FAO/IAEA/USDA 2014) that could undermine the success of a management program. Through our single-pair crosses we determined the CI levels of *w*Ha and *w*Tei lines and showed that *w*Ha induces strong CI in *D. suzukii*, but *w*Tei CI levels are rather moderate. The *w*Tei results are not in alignment with the study performed by Cattel et al. (2018), in which a high level of CI was induced by the *w*Tei-infected *D. suzukii* line (96.46% CI_corr_ for the uninfected females). The observed differences could be due to the alternate host genetic background used in the two studies. These effects might be proved to be detrimental for the biological quality of the host (Calvitti et al. 2010; Fraser et al. 2017), and the cost–benefit evaluation will guide the decision for the use of a specific strain in large-scale applications. The profound influence of the *D. suzukii* genetic background on the CI expression indicates that the host–bacterial symbiotic association should be first and foremost characterized before a potential IIT application. As a result, the SIT presents an advantage over the IIT on the grounds that in the SIT the insects from the targeted field population can be mass-reared, sterilized and released in the area, thus surpassing the barrier of the host genetic background. However, it is worth noting that SIT studies on *D. suzukii* that tested high irradiation doses on flies with different genetic backgrounds provided comparable results (Krüger et al. 2018; Lanouette et al. 2017; Sassù et  al. 2019). In the study of Krüger and colleagues, the flies were collected in Brazil while the other two studies used flies from the same colony reared at the IPCL (Italian genetic background). Despite the wide range of irradiation doses tested in all the above studies, the results did not show any major discrepancies both for males and females.

Young males can induce high CI levels but the increasing age exhibits an apparent and rapid decline in CI expression (Awrahman et al. 2014; Reynolds and Hoffmann 2002). This was also confirmed by our study and in the case of *w*Ha, young males aged 2–3 days old seem to have a better performance in exhibiting a high CI profile, while the CI levels induced by *w*Tei males aged 2–3 days and 5–6 days old do not vary. In our irradiation experiments, the *w*Ha and *w*Tei males were 5–6 days old compared to the 2-to-3-day-old males used in the CI experiments. The low egg hatching we observed in the combined irradiation/*Wolbachia*-infected cases (Table 1) could be explained by the male age that seems to negatively affect the CI_corr_ levels. Especially concerning the *w*Tei males, none of the three irradiation doses tested were free of “escapers” that reached the adult stage, while in the case of the *w*Ha males none of the hatched eggs reached the adult stage. The male age is a factor that should be considered in the logistics burden of any operational program with an IIT component for *D. suzukii*, since only young males that exhibit high CI levels should be released on a frequent basis.

*Drosophila suzukii* constitutes a continuously expanding threat and its exceptional biological traits have elevated the management of this pest into a challenge. The absence of a robust and adequate sexing system for *D. suzukii* renders IIT, as a stand-alone control method, an unattainable choice (Nikolouli et al. 2018). The currently available studies performed on SIT and *D. suzukii* by using irradiation in the range of 120–200 Gy are in favor of the feasibility of this approach. Further knowledge should be acquired regarding the male mating competitiveness, the longevity and the flight ability of the sterile males, particularly in the field, prior to its deployment (Vreysen et al. 2006; Zhang et al. 2016; Parker and Mehta 2007). As shown in the mosquito vector species *Ae. albopictus*, combining the SIT with IIT has the advantage of requiring significantly reduced levels of radiation which may not significantly affect the biological quality of the sterile males (Zhang et al. 2016; Zheng et al. 2019). The combined SIT/IIT also presents an advantage over the IIT since the radiation-induced sterility complements the one induced by *Wolbachia* infection. Based on these as well as on the data presented in this study where a range of 45–90 Gy irradiation doses was tested, the combined SIT/IIT may also worth consideration as an alternative approach for the population suppression of *D. suzukii* (Nikolouli et al. 2018). However, knowledge on male mating competitiveness will also be required prior to any small or large-scale application.

## Author contribution statement

KN and KB designed the study. KN performed the experiments, did the data analysis and wrote the manuscript. All authors reviewed and provided constructive comments for this manuscript. All authors read and approved the final manuscript.

## Electronic supplementary material

Below is the link to the electronic supplementary material.

**Online Resource 1** Effect of *Wolbachia* infection status on *D. suzukii***a** fecundity and **b** hatch rate. A GLMM (binomial family) analysis was performed to determine the differences among the groups for fecundity (Poisson family) and hatch rate (binomial family), but differences were not significant. **Online Resource 2** Effect of *Wolbachia* infection status on *D. suzukii* pupal weight. The different letter codes indicate statistically significant differences between lines. A linear mixed-effect model analysis (ANOVA followed by Tukey’s test) was performed to determine the differences among the lines. **Online Resource 3** Effect of *Wolbachia* infection status on *D. suzukii***a** female and **b** male longevity. Flies were provided with standard rearing diet and dead flies were recorded daily. The x-axis represents time in days. Significant differences were measured with a log-rank test. **Online Resource 4** Statistical analysis results of all datasets (DOCX 376 kb)
