# Supplementary material for: Combining sterile and incompatible insect techniques for the population suppression of Drosophila suzukii
Source: J Pest Sci (2004). 2020 Jan 29;93(2):647–61. doi: 10.1007/s10340-020-01199-6 (PMC7028798; doi:10.1007/s10340-020-01199-6)
Supplement: Supplementary file 1 — Online Resource 1 Effect of Wolbachia infection status on D. suzukiia fecundity and b hatch rate. A GLMM (binomial family) analysis was performed to determine the differences among the groups for fecundity (Poisson family) and hatch rate (binomial family), but differences were not significant. Online Resource 2 Effect of Wolbachia infection status on D. suzukii pupal weight. The different letter codes indicate statistically significant differences between lines. A linear mixed-effect model analysis (ANOVA followed by Tukey’s test) was performed to determine the differences among the lines. Online Resource 3 Effect of Wolbachia infection status on D. suzukiia female and b male longevity. Flies were provided with standard rearing diet and dead flies were recorded daily. The x-axis represents time in days. Significant differences were measured with a log-rank test. Online Resource 4 Statistical analysis results of all datasets (DOCX 376 kb) [file 10340_2020_1199_MOESM1_ESM.docx]

**Combining sterile and incompatible insect techniques for the population suppression of**

***Drosophila suzukii***

Journal of Pest Science

Nikolouli K., Sassù F., Mouton L., Stauffer C., Bourtzis K.*

*Insect Pest Control Section, Joint FAO/IAEA Division of Nuclear Techniques in Food and Agriculture, Wagramerstrasse 5, PO Box 100, A-1400 Vienna, Austria

*Corresponding author: Kostas Bourtzis, [K.Bourtzis@iaea.org](mailto:K.Bourtzis@iaea.org)

**
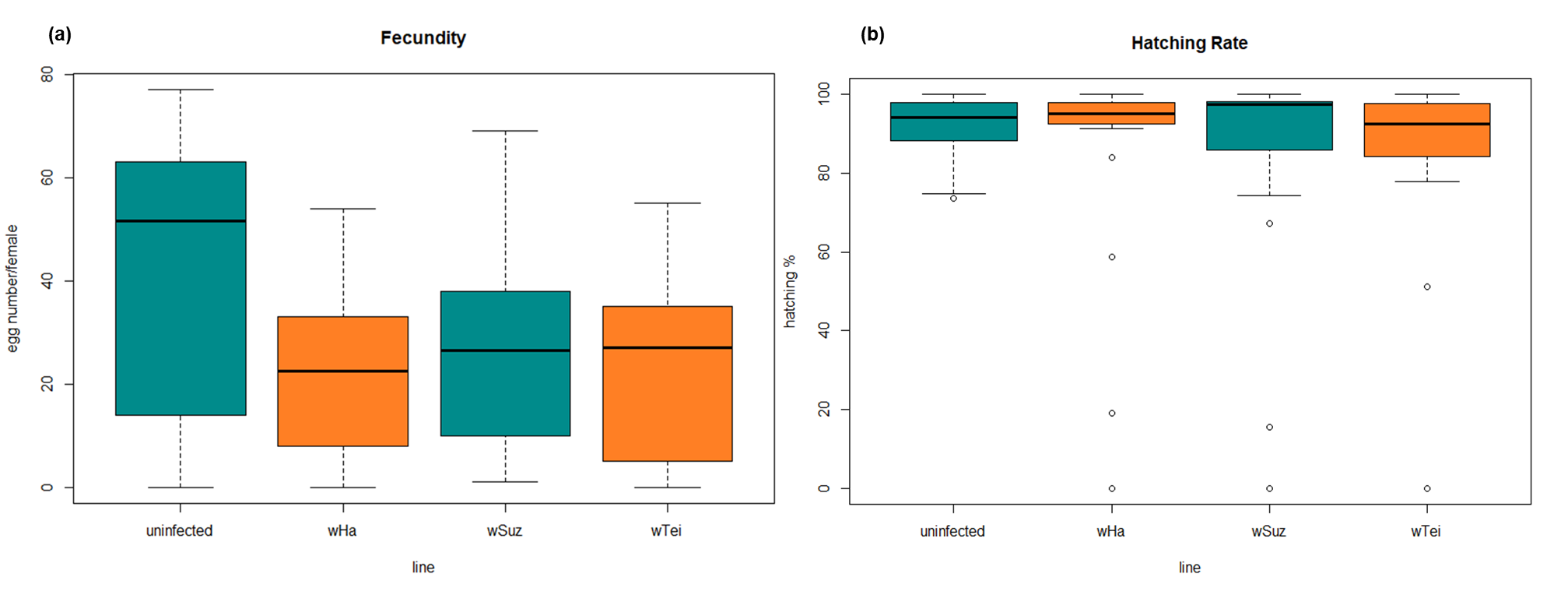
**

**Online Resource 1**


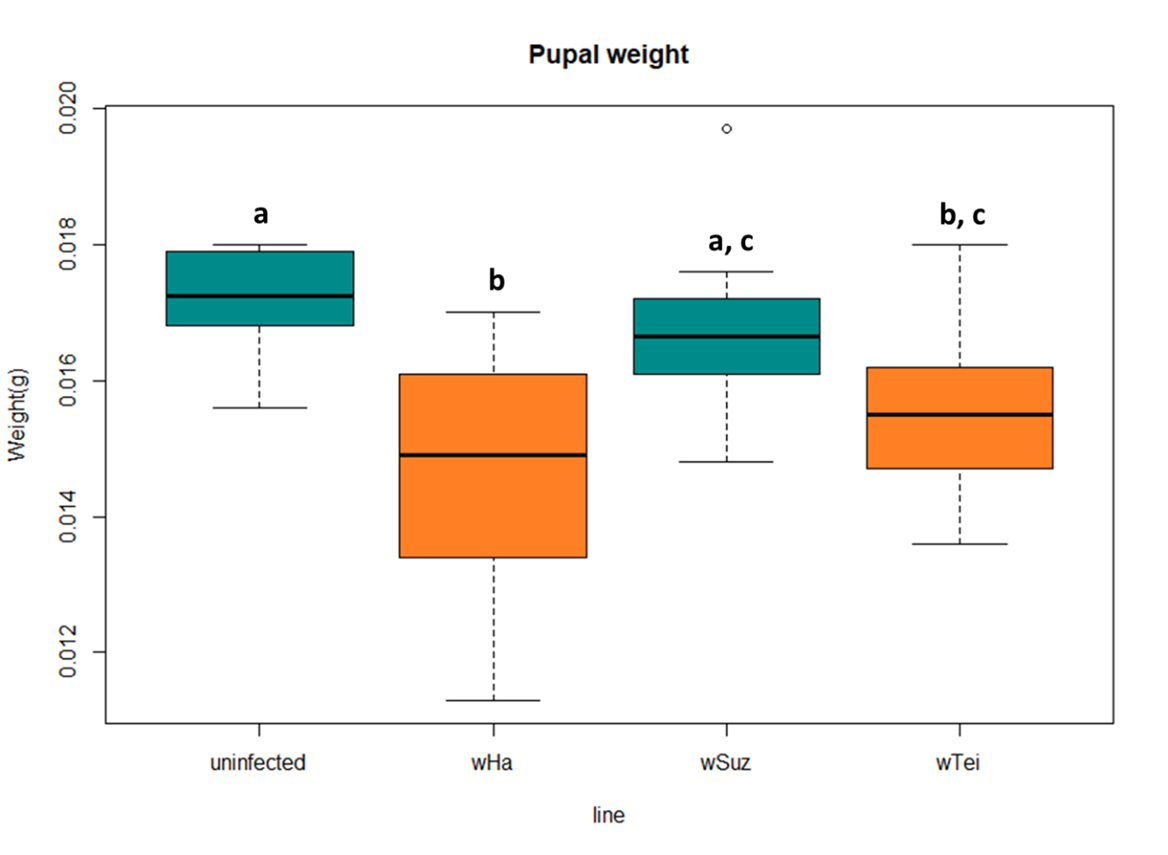


**Online Resource 2**

**Online Resource 3**


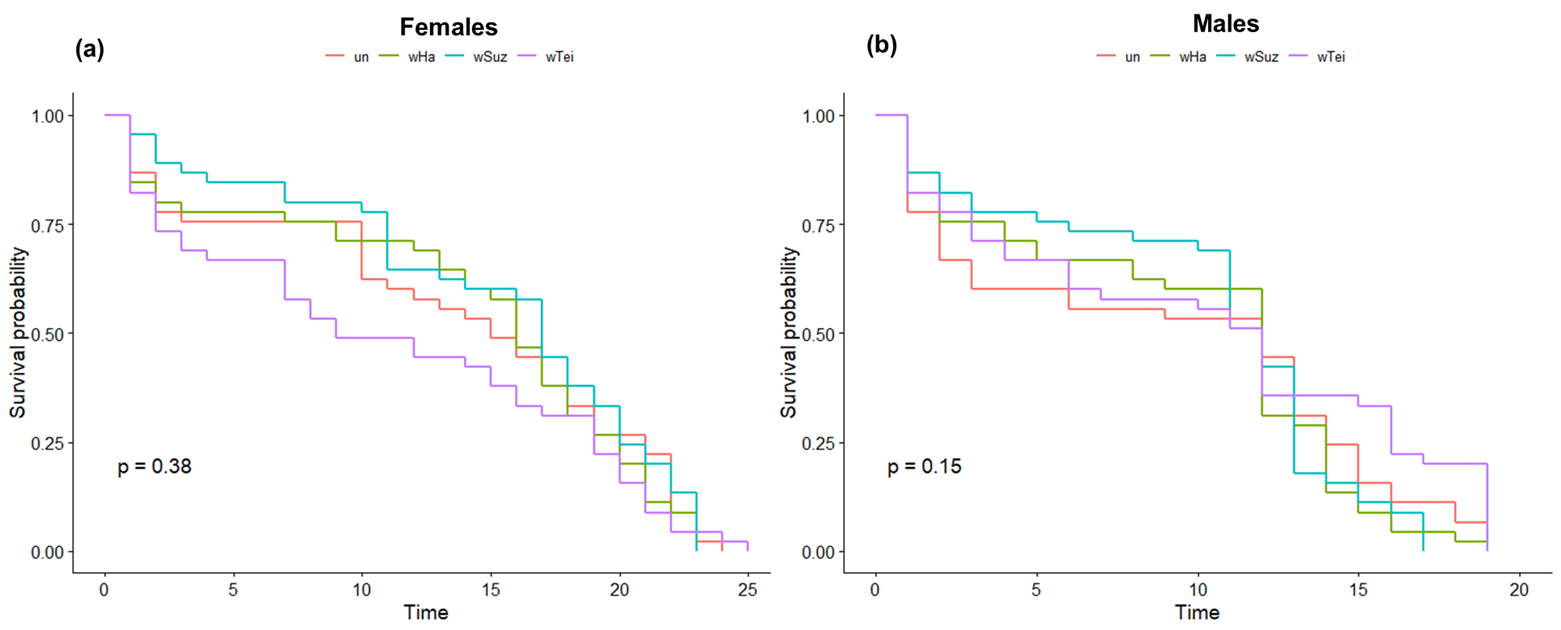


**Online Resource 4**

**Statistical results of all datasets**

**####** **Fecundity**

> ###summary statistics for fecundity###

> library(dplyr)

> group_by(fec2, line2) %>%

+ summarise(

+ count = n(),

+ mean = mean(eggs, na.rm = TRUE),

+ sd = sd(eggs, na.rm = TRUE),

+ median = median(eggs, na.rm = TRUE),

+ IQR = IQR(eggs, na.rm = TRUE))

# A tibble: 4 x 6

line2 count mean sd median IQR

*<fct>* *<int>* *<dbl>* *<dbl>* *<dbl>* *<dbl>*

1 un 30 42.7 25.1 51.5 48.8

2 wHa 30 22.7 15.7 22.5 22.5

3 wSuz 30 26 18.2 26.5 27.8

4 wTei 30 22 15.9 27 28.8

> ###GLMM for fecundity###

> library(lme4)

> m1 <- glmer(eggs~line2+(1|batch2/ind2), family=poisson, data=fec2)

> summary(m1)

Generalized linear mixed model fit by maximum likelihood (Laplace Approximation) ['glmerMod']

Family: poisson ( log )

Formula: eggs ~ line2 + (1 | batch2/ind2)

Data: fec2

AIC BIC logLik deviance df.resid

1081.2 1097.9 -534.6 1069.2 114

Scaled residuals:

Min 1Q Median 3Q Max

-1.69017 -0.11336 0.05105 0.10682 0.23621

Random effects:

Groups Name Variance Std.Dev.

ind2:batch2 (Intercept) 0.9174 0.9578

batch2 (Intercept) 0.1828 0.4275

Number of obs: 120, groups: ind2:batch2, 120; batch2, 12

Fixed effects:

Estimate Std. Error z value Pr(>|z|)

(Intercept) 3.4172 0.3054 11.189 <2e-16 ***

line2wHa -0.6977 0.4338 -1.608 0.1078

line2wSuz -0.4728 0.4324 -1.093 0.2742

line2wTei -0.7963 0.4345 -1.833 0.0668 .

---

Signif. codes: 0 ‘***’ 0.001 ‘**’ 0.01 ‘*’ 0.05 ‘.’ 0.1 ‘ ’ 1

Correlation of Fixed Effects:

(Intr) lin2wH ln2wSz

line2wHa -0.703

line2wSuz -0.706 0.497

line2wTei -0.702 0.496 0.496

> anova(m1, test='Chi')

Analysis of Variance Table

Df Sum Sq Mean Sq F value

line2 3 4.0161 1.3387 1.3387

> library(blmeco)

> dispersion_glmer(m1)

[1] 1.1316

###group comparisons for fecundity###

> library(multcomp)

> multiple_test1=summary(glht(m1,mcp(line2="Tukey")))

> summary(multiple_test1)

Simultaneous Tests for General Linear Hypotheses

Multiple Comparisons of Means: Tukey Contrasts

Fit: glmer(formula = eggs ~ line2 + (1 | batch2/ind2), data = fec2,

family = poisson)

Linear Hypotheses:

Estimate Std. Error z value Pr(>|z|)

wHa - un == 0 -0.69768 0.43383 -1.608 0.374

wSuz - un == 0 -0.47281 0.43244 -1.093 0.694

wTei - un == 0 -0.79627 0.43448 -1.833 0.258

wSuz - wHa == 0 0.22487 0.43443 0.518 0.955

wTei - wHa == 0 -0.09859 0.43603 -0.226 0.996

wTei - wSuz == 0 -0.32346 0.43510 -0.743 0.879

(Adjusted p values reported -- single-step method)

> cld(multiple_test1)

un wHa wSuz wTei

"a" "a" "a" "a"

**###Pupae weight**

> ###summary statistics for pupae weight###

> library(dplyr)

> group_by(pup2, line2) %>%

+ summarise(

+ count = n(),

+ mean = mean(weight, na.rm = TRUE),

+ sd = sd(weight, na.rm = TRUE),

+ median = median(weight, na.rm = TRUE),

+ IQR = IQR(weight, na.rm = TRUE))

# A tibble: 4 x 6

line2 count mean sd median IQR

*<fct>* *<int>* *<dbl>* *<dbl>* *<dbl>* *<dbl>*

1 uninfected 10 0.0172 0.000781 0.0173 0.001

2 wHa 10 0.0147 0.00166 0.0149 0.00230

3 wSuz 10 0.0167 0.00137 0.0166 0.00105

4 wTei 10 0.0155 0.00126 0.0155 0.0014

> ###check for normality of data with Shapiro test###

> shapiro.test(pup2$weight)

Shapiro-Wilk normality test

data: pup2$weight

W = 0.97442, p-value = 0.4908

###Linear model for pupae weight###

> m5 <- lmer((weight)^2~line2 + (1|gen2:rep2), data=pup2, na.action = na.exclude)

> summary(m5)

Linear mixed model fit by REML ['lmerMod']

Formula: (weight)^2 ~ line2 + (1 | gen2:rep2)

Data: pup2

REML criterion at convergence: -616.8

Scaled residuals:

Min 1Q Median 3Q Max

-1.90591 -0.49503 -0.03229 0.51873 2.34973

Random effects:

Groups Name Variance Std.Dev.

gen2:rep2 (Intercept) 5.760e-10 2.400e-05

Residual 1.146e-09 3.385e-05

Number of obs: 40, groups: gen2:rep2, 26

Fixed effects:

Estimate Std. Error t value

(Intercept) 2.947e-04 1.312e-05 22.455

line2wHa -7.003e-05 1.836e-05 -3.815

line2wSuz -1.815e-05 1.843e-05 -0.985

line2wTei -5.805e-05 1.843e-05 -3.151

Correlation of Fixed Effects:

(Intr) lin2wH ln2wSz

line2wHa -0.715

line2wSuz -0.712 0.582

line2wTei -0.712 0.582 0.662

###group comparisons###

> library(emmeans)

> d5 <- emmeans(m5, list(pairwise ~ line2), adjust = "tukey")

> d5

$`emmeans of line2`

line2 emmean SE df asymp.LCL asymp.UCL

uninfected 0.000295 1.31e-05 Inf 0.000269 0.000320

wHa 0.000225 1.28e-05 Inf 0.000199 0.000250

wSuz 0.000277 1.29e-05 Inf 0.000251 0.000302

wTei 0.000237 1.29e-05 Inf 0.000211 0.000262

Degrees-of-freedom method: asymptotic

Results are given on the ( (not the response) scale.

Confidence level used: 0.95

$`pairwise differences of line2`

contrast estimate SE df z.ratio p.value

uninfected - wHa 7.00e-05 1.84e-05 Inf 3.815 0.0008

uninfected - wSuz 1.82e-05 1.84e-05 Inf 0.985 0.7580

uninfected - wTei 5.81e-05 1.84e-05 Inf 3.151 0.0088

wHa - wSuz -5.19e-05 1.68e-05 Inf -3.084 0.0110

wHa - wTei -1.20e-05 1.68e-05 Inf -0.712 0.8925

wSuz - wTei 3.99e-05 1.51e-05 Inf 2.636 0.0418

P value adjustment: tukey method for comparing a family of 4 estimates

**###Sex ratio_females**

###summary statistics for females###

> library(dplyr)

> group_by(sexf, line2) %>%

+ summarise(

+ count = n(),

+ mean = mean(females/progeny, na.rm = TRUE),

+ sd = sd(females/progeny, na.rm = TRUE),

+ median = median(females/progeny, na.rm = TRUE),

+ IQR = IQR(females/progeny, na.rm = TRUE))

# A tibble: 4 x 6

line2 count mean sd median IQR

*<fct>* *<int>* *<dbl>* *<dbl>* *<dbl>* *<dbl>*

1 uninfected 22 0.513 0.122 0.494 0.169

2 wHa 26 0.494 0.149 0.5 0.182

3 wSuz 24 0.443 0.0874 0.435 0.133

4 wTei 28 0.502 0.140 0.512 0.157

###GLMM for females ratio###

> library(lme4)

> m2 <- glmer(females~line2 + (1|batch2/rep2), data=sexf, family = "poisson")

> summary(m2)

Generalized linear mixed model fit by maximum likelihood (Laplace Approximation) ['glmerMod']

Family: poisson ( log )

Formula: females ~ line2 + (1 | batch2/rep2)

Data: sexf

AIC BIC logLik deviance df.resid

688.6 704.3 -338.3 676.6 94

Scaled residuals:

Min 1Q Median 3Q Max

-2.01424 -0.40873 0.03061 0.27452 0.87665

Random effects:

Groups Name Variance Std.Dev.

rep2:batch2 (Intercept) 0.2162 0.465

batch2 (Intercept) 0.1183 0.344

Number of obs: 100, groups: rep2:batch2, 100; batch2, 12

Fixed effects:

Estimate Std. Error z value Pr(>|z|)

(Intercept) 2.56991 0.23049 11.150 <2e-16 ***

line2wHa -0.26448 0.32387 -0.817 0.414

line2wSuz -0.24838 0.32535 -0.763 0.445

line2wTei -0.09117 0.32154 -0.284 0.777

---

Signif. codes: 0 ‘***’ 0.001 ‘**’ 0.01 ‘*’ 0.05 ‘.’ 0.1 ‘ ’ 1

Correlation of Fixed Effects:

(Intr) lin2wH ln2wSz

line2wHa -0.710

line2wSuz -0.707 0.504

line2wTei -0.715 0.510 0.507

> anova(m2, test='Chi')

Analysis of Variance Table

Df Sum Sq Mean Sq F value

line2 3 0.92856 0.30952 0.3095

> library(blmeco)

> dispersion_glmer(m2)

[1] 1.085412

###group comparisons for females ratio###

> library(multcomp)

> multiple_test2=summary(glht(m2,mcp(line2="Tukey")))

> multiple_test2

Simultaneous Tests for General Linear Hypotheses

Multiple Comparisons of Means: Tukey Contrasts

Fit: glmer(formula = females ~ line2 + (1 | batch2/rep2), data = sexf,

family = "poisson")

Linear Hypotheses:

Estimate Std. Error z value Pr(>|z|)

wHa - uninfected == 0 -0.26448 0.32387 -0.817 0.847

wSuz - uninfected == 0 -0.24838 0.32535 -0.763 0.871

wTei - uninfected == 0 -0.09117 0.32154 -0.284 0.992

wSuz - wHa == 0 0.01610 0.32344 0.050 1.000

wTei - wHa == 0 0.17332 0.31961 0.542 0.949

wTei - wSuz == 0 0.15722 0.32113 0.490 0.961

(Adjusted p values reported -- single-step method)

> cld(multiple_test2)

uninfected wHa wSuz wTei

"a" "a" "a" "a"

**###Sex ratio_males**

###summary statistics for males###

> library(dplyr)

> group_by(sexm, line2) %>%

+ summarise(

+ count = n(),

+ mean = mean(males/progeny, na.rm = TRUE),

+ sd = sd(males/progeny, na.rm = TRUE),

+ median = median(males/progeny, na.rm = TRUE),

+ IQR = IQR(males/progeny, na.rm = TRUE))

# A tibble: 4 x 6

line2 count mean sd median IQR

*<fct>* *<int>* *<dbl>* *<dbl>* *<dbl>* *<dbl>*

1 uninfected 22 0.487 0.122 0.506 0.169

2 wHa 26 0.506 0.149 0.5 0.182

3 wSuz 24 0.557 0.0874 0.565 0.133

4 wTei 28 0.498 0.140 0.488 0.157

###GLMM for males ratio###

> library(lme4)

> m2 <- glmer(males~line2 + (1|batch2/rep2), data=sexm, family = "poisson")

> summary(m2)

Generalized linear mixed model fit by maximum likelihood (Laplace Approximation) ['glmerMod']

Family: poisson ( log )

Formula: males ~ line2 + (1 | batch2/rep2)

Data: sexm

AIC BIC logLik deviance df.resid

681.1 696.7 -334.5 669.1 94

Scaled residuals:

Min 1Q Median 3Q Max

-1.53840 -0.52064 0.04031 0.33063 0.69677

Random effects:

Groups Name Variance Std.Dev.

rep2:batch2 (Intercept) 0.2180 0.4669

batch2 (Intercept) 0.1181 0.3437

Number of obs: 100, groups: rep2:batch2, 100; batch2, 12

Fixed effects:

Estimate Std. Error z value Pr(>|z|)

(Intercept) 2.52120 0.23130 10.900 <2e-16 ***

line2wHa -0.24734 0.32441 -0.762 0.446

line2wSuz -0.02719 0.32471 -0.084 0.933

line2wTei -0.12441 0.32249 -0.386 0.700

---

Signif. codes: 0 ‘***’ 0.001 ‘**’ 0.01 ‘*’ 0.05 ‘.’ 0.1 ‘ ’ 1

Correlation of Fixed Effects:

(Intr) lin2wH ln2wSz

line2wHa -0.711

line2wSuz -0.711 0.506

line2wTei -0.716 0.510 0.510

> anova(m2)

Analysis of Variance Table

Df Sum Sq Mean Sq F value

line2 3 0.71869 0.23956 0.2396

> library(blmeco)

> dispersion_glmer(m2)

[1] 1.020424

###group comparisons for males ratio###

> library(multcomp)

> multiple_test2=summary(glht(m2,mcp(line2="Tukey")))

> multiple_test2

Simultaneous Tests for General Linear Hypotheses

Multiple Comparisons of Means: Tukey Contrasts

Fit: glmer(formula = males ~ line2 + (1 | batch2/rep2), data = sexm,

family = "poisson")

Linear Hypotheses:

Estimate Std. Error z value Pr(>|z|)

wHa - uninfected == 0 -0.24734 0.32441 -0.762 0.871

wSuz - uninfected == 0 -0.02719 0.32471 -0.084 1.000

wTei - uninfected == 0 -0.12441 0.32249 -0.386 0.980

wSuz - wHa == 0 0.22015 0.32248 0.683 0.904

wTei - wHa == 0 0.12293 0.32019 0.384 0.981

wTei - wSuz == 0 -0.09722 0.32050 -0.303 0.990

(Adjusted p values reported -- single-step method)

> cld(multiple_test2)

uninfected wHa wSuz wTei

"a" "a" "a" "a"

**###Hatch rate**

###summary statistics for hatch rate###

> library(dplyr)

> group_by(hatch, line2) %>%

+ summarise(

+ count = n(),

+ mean = mean(hatch_rate, na.rm = TRUE),

+ sd = sd(hatch_rate, na.rm = TRUE),

+ median = median(hatch_rate, na.rm = TRUE),

+ IQR = IQR(hatch_rate, na.rm = TRUE))

# A tibble: 4 x 6

line2 count mean sd median IQR

*<fct>* *<int>* *<dbl>* *<dbl>* *<dbl>* *<dbl>*

1 uninfected 24 91.5 8.19 94.2 9.43

2 wHa 21 86.0 27.1 95.1 5.37

3 wSuz 20 84.5 27.8 97.4 11.3

4 wTei 21 77.9 34.3 92.5 13.3

###GLMM model for hatch rate###

> m2 <- glmer(hatched2~line2+(1|replicate:eggs), data = hatch2, family = "binomial")

Warning messages:

1: In checkConv(attr(opt, "derivs"), opt$par, ctrl = control$checkConv, :

unable to evaluate scaled gradient

2: In checkConv(attr(opt, "derivs"), opt$par, ctrl = control$checkConv, :

Model failed to converge: degenerate Hessian with 1 negative eigenvalues

> ss <- getME(m2,c("theta","fixef"))

> m3 <- update(m2,start=ss,control=glmerControl(optCtrl=list(maxfun=2e4)))

> summary(m3)

Generalized linear mixed model fit by maximum likelihood (Laplace Approximation) ['glmerMod']

Family: binomial ( logit )

Formula: hatched2 ~ line2 + (1 | replicate:eggs)

Data: hatch2

Control: glmerControl(optCtrl = list(maxfun = 20000))

AIC BIC logLik deviance df.resid

1693.6 1724.8 -841.8 1683.6 3795

Scaled residuals:

Min 1Q Median 3Q Max

-0.087633 0.001735 0.001987 0.002404 0.002750

Random effects:

Groups Name Variance Std.Dev.

replicate:eggs (Intercept) 2306 48.02

Number of obs: 3800, groups: replicate:eggs, 3800

Fixed effects:

Estimate Std. Error z value Pr(>|z|)

(Intercept) 12.7070 0.6044 21.023 <2e-16 ***

line2wHa -0.6594 0.7277 -0.906 0.365

line2wSuz -0.2739 0.7904 -0.347 0.729

line2wTei -0.9322 0.7207 -1.294 0.196

---

Signif. codes: 0 ‘***’ 0.001 ‘**’ 0.01 ‘*’ 0.05 ‘.’ 0.1 ‘ ’ 1

Correlation of Fixed Effects:

(Intr) lin2wH ln2wSz

line2wHa -0.767

line2wSuz -0.705 0.587

line2wTei -0.773 0.642 0.592

> library(blmeco)

> dispersion_glmer(m3)

[1] 0.1420725

###group comparisons for hatch rate###

> library(multcomp)

> multiple_test=summary(glht(m3,mcp(line2="Tukey")))

> multiple_test

Simultaneous Tests for General Linear Hypotheses

Multiple Comparisons of Means: Tukey Contrasts

Fit: glmer(formula = hatched2 ~ line2 + (1 | replicate:eggs), data = hatch2,

family = "binomial", control = glmerControl(optCtrl = list(maxfun = 20000)),

start = ss)

Linear Hypotheses:

Estimate Std. Error z value Pr(>|z|)

wHa - un == 0 -0.6594 0.7277 -0.906 0.800

wSuz - un == 0 -0.2739 0.7904 -0.347 0.986

wTei - un == 0 -0.9322 0.7207 -1.294 0.565

wSuz - wHa == 0 0.3855 0.6925 0.557 0.944

wTei - wHa == 0 -0.2728 0.6127 -0.445 0.970

wTei - wSuz == 0 -0.6583 0.6857 -0.960 0.771

(Adjusted p values reported -- single-step method)

> cld(multiple_test)

un wHa wSuz wTei

"a" "a" "a" "a"

**##Adult longevity**

###Females###

> library(survival)

> mfit <- survfit(Surv(time=longf2$lifespan, event=longf2$event) ~ line, data = longf2)

> mfit

Call: survfit(formula = Surv(time = longf2$lifespan, event = longf2$event) ~

line, data = longf2)

n events median 0.95LCL 0.95UCL

line=un 45 45 15 10 19

line=wHa 45 45 16 14 18

line=wSuz 45 45 17 13 20

line=wTei 45 45 9 7 17

> summary(mfit)

Call: survfit(formula = Surv(time = longf2$lifespan, event = longf2$event) ~

line, data = longf2)

line=un

time n.risk n.event survival std.err lower 95% CI upper 95% CI

1 45 6 0.8667 0.0507 0.7728 0.972

2 39 4 0.7778 0.0620 0.6653 0.909

3 35 1 0.7556 0.0641 0.6399 0.892

10 34 6 0.6222 0.0723 0.4955 0.781

11 28 1 0.6000 0.0730 0.4727 0.762

12 27 1 0.5778 0.0736 0.4501 0.742

13 26 1 0.5556 0.0741 0.4278 0.721

14 25 1 0.5333 0.0744 0.4058 0.701

15 24 2 0.4889 0.0745 0.3626 0.659

16 22 2 0.4444 0.0741 0.3206 0.616

17 20 3 0.3778 0.0723 0.2597 0.550

18 17 2 0.3333 0.0703 0.2205 0.504

19 15 3 0.2667 0.0659 0.1643 0.433

21 12 2 0.2222 0.0620 0.1286 0.384

22 10 6 0.0889 0.0424 0.0349 0.227

23 4 3 0.0222 0.0220 0.0032 0.154

24 1 1 0.0000 NaN NA NA

line=wHa

time n.risk n.event survival std.err lower 95% CI upper 95% CI

1 45 7 0.8444 0.0540 0.7449 0.957

2 38 2 0.8000 0.0596 0.6913 0.926

3 36 1 0.7778 0.0620 0.6653 0.909

7 35 1 0.7556 0.0641 0.6399 0.892

9 34 2 0.7111 0.0676 0.5903 0.857

12 32 1 0.6889 0.0690 0.5661 0.838

13 31 2 0.6444 0.0714 0.5187 0.801

14 29 2 0.6000 0.0730 0.4727 0.762

15 27 1 0.5778 0.0736 0.4501 0.742

16 26 5 0.4667 0.0744 0.3415 0.638

17 21 4 0.3778 0.0723 0.2597 0.550

18 17 3 0.3111 0.0690 0.2014 0.481

19 14 2 0.2667 0.0659 0.1643 0.433

20 12 3 0.2000 0.0596 0.1115 0.359

21 9 4 0.1111 0.0468 0.0486 0.254

22 5 1 0.0889 0.0424 0.0349 0.227

23 4 4 0.0000 NaN NA NA

line=wSuz

time n.risk n.event survival std.err lower 95% CI upper 95% CI

1 45 2 0.956 0.0307 0.8972 1.000

2 43 3 0.889 0.0468 0.8017 0.986

3 40 1 0.867 0.0507 0.7728 0.972

4 39 1 0.844 0.0540 0.7449 0.957

7 38 2 0.800 0.0596 0.6913 0.926

10 36 1 0.778 0.0620 0.6653 0.909

11 35 6 0.644 0.0714 0.5187 0.801

13 29 1 0.622 0.0723 0.4955 0.781

14 28 1 0.600 0.0730 0.4727 0.762

16 27 1 0.578 0.0736 0.4501 0.742

17 26 6 0.444 0.0741 0.3206 0.616

18 20 3 0.378 0.0723 0.2597 0.550

19 17 2 0.333 0.0703 0.2205 0.504

20 15 4 0.244 0.0641 0.1462 0.409

21 11 2 0.200 0.0596 0.1115 0.359

22 9 3 0.133 0.0507 0.0633 0.281

23 6 6 0.000 NaN NA NA

line=wTei

time n.risk n.event survival std.err lower 95% CI upper 95% CI

1 45 8 0.8222 0.0570 0.7178 0.942

2 37 4 0.7333 0.0659 0.6149 0.875

3 33 2 0.6889 0.0690 0.5661 0.838

4 31 1 0.6667 0.0703 0.5422 0.820

7 30 4 0.5778 0.0736 0.4501 0.742

8 26 2 0.5333 0.0744 0.4058 0.701

9 24 2 0.4889 0.0745 0.3626 0.659

12 22 2 0.4444 0.0741 0.3206 0.616

14 20 1 0.4222 0.0736 0.3000 0.594

15 19 2 0.3778 0.0723 0.2597 0.550

16 17 2 0.3333 0.0703 0.2205 0.504

17 15 1 0.3111 0.0690 0.2014 0.481

19 14 4 0.2222 0.0620 0.1286 0.384

20 10 3 0.1556 0.0540 0.0787 0.307

21 7 3 0.0889 0.0424 0.0349 0.227

22 4 2 0.0444 0.0307 0.0115 0.172

24 2 1 0.0222 0.0220 0.0032 0.154

25 1 1 0.0000 NaN NA NA

> mdiff.comp<- survdiff(Surv(time=longf2$lifespan, event=longf2$event)~ line, data = longf2)

> mdiff.comp

Call:

survdiff(formula = Surv(time = longf2$lifespan, event = longf2$event) ~

line, data = longf2)

N Observed Expected (O-E)^2/E (O-E)^2/V

line=un 45 45 46.6 0.0562 0.0940

line=wHa 45 45 44.3 0.0127 0.0206

line=wSuz 45 45 51.7 0.8586 1.5001

line=wTei 45 45 37.5 1.5126 2.3887

Chisq= 3.1 on 3 degrees of freedom, p= 0.4

###Males###

> library(survival)

> mfit <- survfit(Surv(time=longm2$lifespan, event=longm2$event) ~ line, data = longm2)

> mfit

Call: survfit(formula = Surv(time = longm2$lifespan, event = longm2$event) ~

line, data = longm2)

n events median 0.95LCL 0.95UCL

line=un 45 45 12 3 13

line=wHa 45 45 12 8 12

line=wSuz 45 45 12 11 13

line=wTei 45 45 12 6 16

> summary(mfit)

Call: survfit(formula = Surv(time = longm2$lifespan, event = longm2$event) ~

line, data = longm2)

line=un

time n.risk n.event survival std.err lower 95% CI upper 95% CI

1 45 10 0.7778 0.0620 0.6653 0.909

2 35 5 0.6667 0.0703 0.5422 0.820

3 30 3 0.6000 0.0730 0.4727 0.762

6 27 2 0.5556 0.0741 0.4278 0.721

9 25 1 0.5333 0.0744 0.4058 0.701

12 24 4 0.4444 0.0741 0.3206 0.616

13 20 6 0.3111 0.0690 0.2014 0.481

14 14 3 0.2444 0.0641 0.1462 0.409

15 11 4 0.1556 0.0540 0.0787 0.307

16 7 2 0.1111 0.0468 0.0486 0.254

18 5 2 0.0667 0.0372 0.0223 0.199

19 3 3 0.0000 NaN NA NA

line=wHa

time n.risk n.event survival std.err lower 95% CI upper 95% CI

1 45 8 0.8222 0.0570 0.7178 0.942

2 37 3 0.7556 0.0641 0.6399 0.892

4 34 2 0.7111 0.0676 0.5903 0.857

5 32 2 0.6667 0.0703 0.5422 0.820

8 30 2 0.6222 0.0723 0.4955 0.781

9 28 1 0.6000 0.0730 0.4727 0.762

12 27 13 0.3111 0.0690 0.2014 0.481

13 14 1 0.2889 0.0676 0.1827 0.457

14 13 7 0.1333 0.0507 0.0633 0.281

15 6 2 0.0889 0.0424 0.0349 0.227

16 4 2 0.0444 0.0307 0.0115 0.172

18 2 1 0.0222 0.0220 0.0032 0.154

19 1 1 0.0000 NaN NA NA

line=wSuz

time n.risk n.event survival std.err lower 95% CI upper 95% CI

1 45 6 0.8667 0.0507 0.7728 0.972

2 39 2 0.8222 0.0570 0.7178 0.942

3 37 2 0.7778 0.0620 0.6653 0.909

5 35 1 0.7556 0.0641 0.6399 0.892

6 34 1 0.7333 0.0659 0.6149 0.875

8 33 1 0.7111 0.0676 0.5903 0.857

10 32 1 0.6889 0.0690 0.5661 0.838

11 31 8 0.5111 0.0745 0.3841 0.680

12 23 4 0.4222 0.0736 0.3000 0.594

13 19 11 0.1778 0.0570 0.0948 0.333

14 8 1 0.1556 0.0540 0.0787 0.307

15 7 2 0.1111 0.0468 0.0486 0.254

16 5 1 0.0889 0.0424 0.0349 0.227

17 4 4 0.0000 NaN NA NA

line=wTei

time n.risk n.event survival std.err lower 95% CI upper 95% CI

1 45 8 0.822 0.0570 0.718 0.942

2 37 2 0.778 0.0620 0.665 0.909

3 35 3 0.711 0.0676 0.590 0.857

4 32 2 0.667 0.0703 0.542 0.820

6 30 3 0.600 0.0730 0.473 0.762

7 27 1 0.578 0.0736 0.450 0.742

10 26 1 0.556 0.0741 0.428 0.721

11 25 2 0.511 0.0745 0.384 0.680

12 23 7 0.356 0.0714 0.240 0.527

15 16 1 0.333 0.0703 0.221 0.504

16 15 5 0.222 0.0620 0.129 0.384

17 10 1 0.200 0.0596 0.111 0.359

19 9 9 0.000 NaN NA NA

> mdiff.comp<- survdiff(Surv(time=longm2$lifespan, event=longm2$event)~ line, data = longm2)

> mdiff.comp

Call:

survdiff(formula = Surv(time = longm2$lifespan, event = longm2$event) ~

line, data = longm2)

N Observed Expected (O-E)^2/E (O-E)^2/V

line=un 45 45 44.5 0.00624 0.0108

line=wHa 45 45 39.6 0.72495 1.1815

line=wSuz 45 45 39.6 0.72393 1.1591

line=wTei 45 45 56.2 2.24800 4.6946

Chisq= 5.2 on 3 degrees of freedom, p= 0.2

**###Cytoplasmic incompatibility (CI) levels**

###summary statistics for CI levels###

> library(dplyr)

> group_by(ci2, cross2) %>%

+ summarise(

+ count = n(),

+ mean = mean(cicorr_per, na.rm = TRUE),

+ sd = sd(cicorr_per, na.rm = TRUE),

+ median = median(cicorr_per, na.rm = TRUE),

+ IQR = IQR(cicorr_per, na.rm = TRUE))

# A tibble: 6 x 6

cross2 count mean sd median IQR

*<fct>* *<int>* *<dbl>* *<dbl>* *<dbl>* *<dbl>*

1 un♀ x un♂ 24 8.54 8.19 5.82 9.43

2 un♀ x wHa♂ 20 98.9 3.07 100 0

3 un♀ x wTei♂ 19 64.8 36.5 79.2 63.8

4 wSuz♀ x wTei♂ 26 67.4 28.6 70.7 39.0

5 wSuz♀ x wHa♂ 22 98.5 3.48 100 0

6 wSuz♀ x wSuz♂ 20 15.5 27.8 2.63 11.3

###GLMM for CI levels###

> m2 <- glmer(cbind(eggs, hatched)~cross2+(1|batch2:rep2), data = ci2, family = "binomial")

> summary(m2)

Generalized linear mixed model fit by maximum likelihood (Laplace Approximation) ['glmerMod']

Family: binomial ( logit )

Formula: cbind(eggs, hatched) ~ cross2 + (1 | batch2:rep2)

Data: ci2

AIC BIC logLik deviance df.resid

768.2 788.3 -377.1 754.2 124

Scaled residuals:

Min 1Q Median 3Q Max

-1.49432 -0.07360 0.00355 0.43575 1.50209

Random effects:

Groups Name Variance Std.Dev.

batch2:rep2 (Intercept) 1.025 1.012

Number of obs: 131, groups: batch2:rep2, 131

Fixed effects:

Estimate Std. Error z value Pr(>|z|)

(Intercept) 0.09475 0.21232 0.446 0.655

cross2un♀ x wHa♂ 5.02355 0.50722 9.904 < 2e-16 ***

cross2un♀ x wTei♂ 1.93199 0.34469 5.605 2.08e-08 ***

cross2wSuz♀ x wTei♂ 1.74313 0.30660 5.685 1.30e-08 ***

cross2wSuz♀ x wHa♂ 4.84485 0.45861 10.564 < 2e-16 ***

cross2wSuz♀ x wSuz♂ 0.18918 0.31653 0.598 0.550

---

Signif. codes: 0 ‘***’ 0.001 ‘**’ 0.01 ‘*’ 0.05 ‘.’ 0.1 ‘ ’ 1

> library(blmeco)

> dispersion_glmer(m2) #result is not >1.4 so NO overdispersion problem

[1] 1.061238

###group comparisons###

> library(multcomp)

> multiple_test=summary(glht(m2,mcp(cross2="Tukey")))

> multiple_test

Simultaneous Tests for General Linear Hypotheses

Multiple Comparisons of Means: Tukey Contrasts

Fit: glmer(formula = cbind(eggs, hatched) ~ cross2 + (1 | batch2:rep2),

data = ci2, family = "binomial")

Linear Hypotheses:

Estimate Std. Error z value Pr(>|z|)

un♀ x wHa♂ - un♀ x un♂ == 0 5.0236 0.5072 9.904 <1e-04 ***

un♀ x wTei♂ - un♀ x un♂ == 0 1.9320 0.3447 5.605 <1e-04 ***

wSuz♀ x wTei♂ - un♀ x un♂ == 0 1.7431 0.3066 5.685 <1e-04 ***

wSuz♀ x wHa♂ - un♀ x un♂ == 0 4.8448 0.4586 10.564 <1e-04 ***

wSuz♀ x wSuz♂ - un♀ x un♂ == 0 0.1892 0.3165 0.598 0.991

un♀ x wTei♂ - un♀ x wHa♂ == 0 -3.0916 0.5140 -6.014 <1e-04 ***

wSuz♀ x wTei♂ - un♀ x wHa♂ == 0 -3.2804 0.4999 -6.562 <1e-04 ***

wSuz♀ x wHa♂ - un♀ x wHa♂ == 0 -0.1787 0.5862 -0.305 1.000

wSuz♀ x wSuz♂ - un♀ x wHa♂ == 0 -4.8344 0.5137 -9.411 <1e-04 ***

wSuz♀ x wTei♂ - un♀ x wTei♂ == 0 -0.1889 0.3429 -0.551 0.994

wSuz♀ x wHa♂ - un♀ x wTei♂ == 0 2.9129 0.4730 6.159 <1e-04 ***

wSuz♀ x wSuz♂ - un♀ x wTei♂ == 0 -1.7428 0.3568 -4.885 <1e-04 ***

wSuz♀ x wHa♂ - wSuz♀ x wTei♂ == 0 3.1017 0.4542 6.829 <1e-04 ***

wSuz♀ x wSuz♂ - wSuz♀ x wTei♂ == 0-1.5539 0.3213 -4.837 <1e-04 ***

wSuz♀ x wSuz♂ - wSuz♀ x wHa♂ == 0 -4.6557 0.4668 -9.973 <1e-04 ***

---

Signif. codes: 0 ‘***’ 0.001 ‘**’ 0.01 ‘*’ 0.05 ‘.’ 0.1 ‘ ’ 1

(Adjusted p values reported -- single-step method)

> cld(multiple_test

un♀ x un♂ "a"

un♀ x wHa♂ "b"

un♀ x wTei♂ "c"

wSuz♀ x wTei♂ "c"

wSuz♀ x wHa♂ "b"

wSuz♀ x wSuz♂ "a"

**###Age of males effect on CI levels**

###summary statistics for age of males effect###

> library(dplyr)

> group_by(male4, cross2, age2) %>%

+ summarise(

+ count = n(),

+ mean = mean(cicorr_per, na.rm = TRUE),

+ sd = sd(cicorr_per, na.rm = TRUE),

+ median = median(cicorr_per, na.rm = TRUE),

+ IQR = IQR(cicorr_per, na.rm = TRUE))

# A tibble: 18 x 7

# Groups: cross2 [6]

cross2 age2 count mean sd median IQR

*<fct>* *<fct>* *<int>* *<dbl>* *<dbl>* *<dbl>* *<dbl>*

1 un♀ x un♂ 2-3d 6 17.8 13.1 17.3 22.7

2 un♀ x un♂ 5-6d 6 9.59 10.3 7.97 7.75

3 un♀ x un♂ 9-10d 6 18.3 15.1 14.1 23.7

4 un♀ x wHa♂ 2-3d 6 97.7 3.07 99.1 2.12

5 un♀ x wHa♂ 5-6d 6 89.2 7.65 88.9 10.4

6 un♀ x wHa♂ 9-10d 6 65.3 27.6 75.2 26.3

7 un♀ x wTei♂ 2-3d 6 56.0 20.3 55.4 19.6

8 un♀ x wTei♂ 5-6d 6 54.5 15.3 57.5 17.3

9 un♀ x wTei♂ 9-10d 6 23.8 8.40 20.9 10.7

10 wSuz♀ x wTei♂ 2-3d 6 53.3 19.3 54.8 29.5

11 wSuz♀ x wTei♂ 5-6d 6 59.8 10.6 60.1 12.9

12 wSuz♀ x wTei♂ 9-10d 6 27.6 18.9 33.2 20.8

13 wSuz♀ x wHa♂ 2-3d 6 96.6 3.21 96.9 4.39

14 wSuz♀ x wHa♂ 5-6d 6 84.0 11.0 86.0 13.8

15 wSuz♀ x wHa♂ 9-10d 6 63.5 32.4 75.7 11.8

16 wSuz♀ x wSuz♂ 2-3d 6 10.6 10.2 7.5 2.61

17 wSuz♀ x wSuz♂ 5-6d 6 7.87 4.21 9.35 6.68

18 wSuz♀ x wSuz♂ 9-10d 6 13.7 7.81 17.5 11.9

###GLMM model for the wHa line###

> library(lme4)

> m1 <- glmer(cbind(eggs, hatched)~male_age_wHa+ (1|batch2:rep2), data = male4, family = "binomial")

Warning message:

In checkConv(attr(opt, "derivs"), opt$par, ctrl = control$checkConv, :

Model failed to converge with max|grad| = 0.00266767 (tol = 0.001, component 1)

> ss <- getME(m1,c("theta","fixef"))

> m1.1 <- update(m1,start=ss)

> summary(m1.1)

Generalized linear mixed model fit by maximum likelihood (Laplace Approximation) ['glmerMod']

Family: binomial ( logit )

Formula: cbind(eggs, hatched) ~ male_age_wHa + (1 | batch2:rep2)

Data: male4

AIC BIC logLik deviance df.resid

564.9 594.5 -269.5 538.9 59

Scaled residuals:

Min 1Q Median 3Q Max

-2.44517 -0.15314 0.00524 0.32990 1.71658

Random effects:

Groups Name Variance Std.Dev.

batch2:rep2 (Intercept) 0.1065 0.3263

Number of obs: 72, groups: batch2:rep2, 72

Fixed effects:

Estimate Std. Error z value Pr(>|z|)

(Intercept) 0.207112 0.145614 1.422 0.155

un♀ x un♂.5-6d -0.095871 0.203894 -0.470 0.638

un♀ x un♂.9-10d 0.004916 0.204673 0.024 0.981

wSuz♀ x wSuz♂.2-3d -0.089154 0.208397 -0.428 0.669

wSuz♀ x wSuz♂.5-6d -0.122989 0.202107 -0.609 0.543

wSuz♀ x wSuz♂.9-10d -0.057855 0.205127 -0.282 0.778

un♀ x wHa♂.2-3d 3.883613 0.313310 12.395 < 2e-16***

un♀ x wHa♂.5-6d 2.287404 0.244721 9.347 < 2e-16***

un♀ x wHa♂.9-10d 1.228728 0.222169 5.531 3.19e-08***

wSuz♀ x wHa♂.2-3d 3.499393 0.317408 11.025 < 2e-16***

wSuz♀ x wHa♂.5-6d 1.861926 0.224331 8.300 < 2e-16***

wSuz♀ x wHa♂.9-10d 1.199206 0.223654 5.362 8.24e-08***

---

Signif. codes: 0 ‘***’ 0.001 ‘**’ 0.01 ‘*’ 0.05 ‘.’ 0.1 ‘ ’ 1

> library(blmeco)

> dispersion_glmer(m1.1) #result is not >1.4 so NO overdispersion problem

[1] 1.156629

###group comparisons for the wHa line###

> library(multcomp)

> multiple_test1=summary(glht(m1.1,mcp(male_age_wHa="Tukey")))

> multiple_test1

Simultaneous Tests for General Linear Hypotheses

Multiple Comparisons of Means: Tukey Contrasts

Fit: glmer(formula = cbind(eggs, hatched) ~ male_age_wHa + (1 | batch2:rep2),

data = male4, family = "binomial", start = ss)

Linear Hypotheses:

Estimate Std. Error z value Pr(>|z|)

un♀ x un♂.5-6d - un♀ x un♂.2-3d == 0 -0.095871 0.203894 -0.470 1.0000

un♀ x un♂.9-10d - un♀ x un♂.2-3d == 0 0.004916 0.204673 0.024 1.0000

wSuz♀ x wSuz♂.2-3d - un♀ x un♂.2-3d == 0 -0.089154 0.208397 -0.428 1.0000

wSuz♀ x wSuz♂.5-6d - un♀ x un♂.2-3d == 0 -0.122989 0.202107 -0.609 1.0000

wSuz♀ x wSuz♂.9-10d - un♀ x un♂.2-3d == 0 -0.057855 0.205127 -0.282 1.0000

un♀ x wHa♂.2-3d - un♀ x un♂.2-3d == 0 3.883613 0.313310 12.395 <0.01 ***

un♀ x wHa♂.5-6d - un♀ x un♂.2-3d == 0 2.287404 0.244721 9.347 <0.01 ***

un♀ x wHa♂.9-10d - un♀ x un♂.2-3d == 0 1.228728 0.222169 5.531 <0.01 ***

wSuz♀ x wHa♂.2-3d - un♀ x un♂.2-3d == 0 3.499393 0.317408 11.025 <0.01 ***

wSuz♀ x wHa♂.5-6d - un♀ x un♂.2-3d == 0 1.861926 0.224331 8.300 <0.01 ***

wSuz♀ x wHa♂.9-10d - un♀ x un♂.2-3d == 0 1.199206 0.223654 5.362 <0.01 ***

un♀ x un♂.9-10d - un♀ x un♂.5-6d == 0 0.100787 0.202633 0.497 1.0000

wSuz♀ x wSuz♂.2-3d - un♀ x un♂.5-6d == 0 0.006717 0.206387 0.033 1.0000

wSuz♀ x wSuz♂.5-6d - un♀ x un♂.5-6d == 0 -0.027118 0.200030 -0.136 1.0000

wSuz♀ x wSuz♂.9-10d - un♀ x un♂.5-6d == 0 0.038016 0.203085 0.187 1.0000

un♀ x wHa♂.2-3d - un♀ x un♂.5-6d == 0 3.979483 0.312077 12.752 <0.01 ***

un♀ x wHa♂.5-6d - un♀ x un♂.5-6d == 0 2.383275 0.243069 9.805 <0.01 ***

un♀ x wHa♂.9-10d - un♀ x un♂.5-6d == 0 1.324599 0.220422 6.009 <0.01 ***

wSuz♀ x wHa♂.2-3d - un♀ x un♂.5-6d == 0 3.595263 0.316122 11.373 <0.01 ***

wSuz♀ x wHa♂.5-6d - un♀ x un♂.5-6d == 0 1.957797 0.222586 8.796 <0.01 ***

wSuz♀ x wHa♂.9-10d - un♀ x un♂.5-6d == 0 1.295077 0.221826 5.838 <0.01 ***

wSuz♀ x wSuz♂.2-3d - un♀ x un♂.9-10d == 0 -0.094070 0.207156 -0.454 1.0000

wSuz♀ x wSuz♂.5-6d - un♀ x un♂.9-10d == 0 -0.127905 0.200830 -0.637 1.0000

wSuz♀ x wSuz♂.9-10d - un♀ x un♂.9-10d == 0 -0.062771 0.203865 -0.308 1.0000

un♀ x wHa♂.2-3d - un♀ x un♂.9-10d == 0 3.878697 0.312377 12.417 <0.01 ***

un♀ x wHa♂.5-6d - un♀ x un♂.9-10d == 0 2.282488 0.243602 9.370 <0.01 ***

un♀ x wHa♂.9-10d - un♀ x un♂.9-10d == 0 1.223812 0.220857 5.541 <0.01 ***

wSuz♀ x wHa♂.2-3d - un♀ x un♂.9-10d == 0 3.494477 0.316562 11.039 <0.01 ***

wSuz♀ x wHa♂.5-6d - un♀ x un♂.9-10d == 0 1.857010 0.223047 8.326 <0.01 ***

wSuz♀ x wHa♂.9-10d - un♀ x un♂.9-10d == 0 1.194290 0.222450 5.369 <0.01 ***

wSuz♀ x wSuz♂.5-6d - wSuz♀ x wSuz♂.2-3d == 0 -0.033835 0.204621 -0.165 1.0000

wSuz♀ x wSuz♂.9-10d - wSuz♀ x wSuz♂.2-3d == 0 0.031299 0.207605 0.151 1.0000

un♀ x wHa♂.2-3d - wSuz♀ x wSuz♂.2-3d == 0 3.972767 0.314931 12.615 <0.01 ***

un♀ x wHa♂.5-6d - wSuz♀ x wSuz♂.2-3d == 0 2.376558 0.246797 9.630 <0.01 ***

un♀ x wHa♂.9-10d - wSuz♀ x wSuz♂.2-3d == 0 1.317882 0.224450 5.872 <0.01 ***

wSuz♀ x wHa♂.2-3d - wSuz♀ x wSuz♂.2-3d == 0 3.588547 0.319013 11.249 <0.01 ***

wSuz♀ x wHa♂.5-6d - wSuz♀ x wSuz♂.2-3d == 0 1.951080 0.226591 8.611 <0.01 ***

wSuz♀ x wHa♂.9-10d - wSuz♀ x wSuz♂.2-3d == 0 1.288360 0.225926 5.703 <0.01 ***

wSuz♀ x wSuz♂.9-10d - wSuz♀ x wSuz♂.5-6d == 0 0.065134 0.201290 0.324 1.0000

un♀ x wHa♂.2-3d - wSuz♀ x wSuz♂.5-6d == 0 4.006602 0.310853 12.889 <0.01 ***

un♀ x wHa♂.5-6d - wSuz♀ x wSuz♂.5-6d == 0 2.410393 0.241537 9.979 <0.01 ***

un♀ x wHa♂.9-10d - wSuz♀ x wSuz♂.5-6d == 0 1.351717 0.218688 6.181 <0.01 ***

wSuz♀ x wHa♂.2-3d - wSuz♀ x wSuz♂.5-6d == 0 3.622382 0.314954 11.501 <0.01 ***

wSuz♀ x wHa♂.5-6d - wSuz♀ x wSuz♂.5-6d == 0 1.984915 0.220878 8.986 <0.01 ***

wSuz♀ x wHa♂.9-10d - wSuz♀ x wSuz♂.5-6d == 0 1.322195 0.220159 6.006 <0.01 ***

un♀ x wHa♂.2-3d - wSuz♀ x wSuz♂.9-10d == 0 3.941467 0.312768 12.602 <0.01 ***

un♀ x wHa♂.5-6d - wSuz♀ x wSuz♂.9-10d == 0 2.345258 0.244037 9.610 <0.01 ***

un♀ x wHa♂.9-10d - wSuz♀ x wSuz♂.9-10d == 0 1.286583 0.221404 5.811 <0.01 ***

wSuz♀ x wHa♂.2-3d - wSuz♀ x wSuz♂.9-10d == 0 3.557247 0.316884 11.226 <0.01 ***

wSuz♀ x wHa♂.5-6d - wSuz♀ x wSuz♂.9-10d == 0 1.919781 0.223576 8.587 <0.01 ***

wSuz♀ x wHa♂.9-10d - wSuz♀ x wSuz♂.9-10d == 0 1.257061 0.222909 5.639 <0.01 ***

un♀ x wHa♂.5-6d - un♀ x wHa♂.2-3d == 0 -1.596209 0.339203 -4.706 <0.01 ***

un♀ x wHa♂.9-10d - un♀ x wHa♂.2-3d == 0 -2.654884 0.322278 -8.238 <0.01 ***

wSuz♀ x wHa♂.2-3d - un♀ x wHa♂.2-3d == 0 -0.384220 0.395109 -0.972 0.9981

wSuz♀ x wHa♂.5-6d - un♀ x wHa♂.2-3d == 0 -2.021687 0.323983 -6.240 <0.01 ***

wSuz♀ x wHa♂.9-10d - un♀ x wHa♂.2-3d == 0 -2.684406 0.324608 -8.270 <0.01 ***

un♀ x wHa♂.9-10d - un♀ x wHa♂.5-6d == 0 -1.058676 0.257436 -4.112 <0.01 **

wSuz♀ x wHa♂.2-3d - un♀ x wHa♂.5-6d == 0 1.211989 0.343586 3.527 0.0201 *

wSuz♀ x wHa♂.5-6d - un♀ x wHa♂.5-6d == 0 -0.425478 0.259423 -1.640 0.8883

wSuz♀ x wHa♂.9-10d - un♀ x wHa♂.5-6d == 0 -1.088197 0.259458 -4.194 <0.01 **

wSuz♀ x wHa♂.2-3d - un♀ x wHa♂.9-10d == 0 2.270664 0.327596 6.931 <0.01 ***

wSuz♀ x wHa♂.5-6d - un♀ x wHa♂.9-10d == 0 0.633198 0.237057 2.671 0.2305

wSuz♀ x wHa♂.9-10d - un♀ x wHa♂.9-10d == 0 -0.029522 0.237864 -0.124 1.0000

wSuz♀ x wHa♂.5-6d - wSuz♀ x wHa♂.2-3d == 0 -1.637467 0.329129 -4.975 <0.01 ***

wSuz♀ x wHa♂.9-10d - wSuz♀ x wHa♂.2-3d == 0 -2.300186 0.328996 -6.992 <0.01 ***

wSuz♀ x wHa♂.9-10d - wSuz♀ x wHa♂.5-6d == 0 -0.662720 0.239974 -2.762 0.1874

---

Signif. codes: 0 ‘***’ 0.001 ‘**’ 0.01 ‘*’ 0.05 ‘.’ 0.1 ‘ ’ 1

(Adjusted p values reported -- single-step method)

> library(emmeans)

> library(lsmeans)

> d <- emmeans(m1.1, "male_age_wHa", type= "response")

> cld(d)

male_age_wHa prob SE df asymp.LCL asymp.UCL .group

wSuz♀ x wSuz♂.5-6d 0.521 0.03498 Inf 0.452 0.589 1

un♀ x un♂.5-6d 0.528 0.03557 Inf 0.458 0.597 1

wSuz♀ x wSuz♂.2-3d 0.529 0.03714 Inf 0.457 0.601 1

wSuz♀ x wSuz♂.9-10d 0.537 0.03592 Inf 0.467 0.606 1

un♀ x un♂.2-3d 0.552 0.03602 Inf 0.480 0.621 1

un♀ x un♂.9-10d 0.553 0.03556 Inf 0.483 0.621 1

wSuz♀ x wHa♂.9-10d 0.803 0.02684 Inf 0.745 0.851 2

un♀ x wHa♂.9-10d 0.808 0.02606 Inf 0.752 0.854 2

wSuz♀ x wHa♂.5-6d 0.888 0.01699 Inf 0.850 0.917 23

un♀ x wHa♂.5-6d 0.924 0.01385 Inf 0.892 0.947 3

wSuz♀ x wHa♂.2-3d 0.976 0.00660 Inf 0.959 0.986 4

un♀ x wHa♂.2-3d 0.984 0.00449 Inf 0.972 0.990 4

Confidence level used: 0.95

Intervals are back-transformed from the logit scale

P value adjustment: tukey method for comparing a family of 12 estimates

Tests are performed on the log odds ratio scale

significance level used: alpha = 0.05

###GLMM model for the wTei line###

> m2 <- glmer(cbind(eggs, hatched)~male_age_wTei+ (1|batch2:rep2), data = male4, family = "binomial")

> summary(m2)

Generalized linear mixed model fit by maximum likelihood (Laplace Approximation) ['glmerMod']

Family: binomial ( logit )

Formula: cbind(eggs, hatched) ~ male_age_wTei + (1 | batch2:rep2)

Data: male4

AIC BIC logLik deviance df.resid

587.8 617.4 -280.9 561.8 59

Scaled residuals:

Min 1Q Median 3Q Max

-1.48703 -0.28508 -0.06956 0.26549 2.70940

Random effects:

Groups Name Variance Std.Dev.

batch2:rep2 (Intercept) 0.03655 0.1912

Number of obs: 72, groups: batch2:rep2, 72

Fixed effects:

Estimate Std. Error z value Pr(>|z|)

(Intercept) 0.206044 0.097385 2.116 0.0344 *

un♀ x un♂.5-6d -0.090081 0.134436 -0.670 0.5028

un♀ x un♂.9-10d -0.001964 0.135603 -0.014 0.9884

wSuz♀ x wSuz♂.2-3d -0.089983 0.140732 -0.639 0.5226

wSuz♀ x wSuz♂.5-6d -0.120755 0.131786 -0.916 0.3595

wSuz♀ x wSuz♂.9-10d -0.059133 0.136624 -0.433 0.6652

un♀ x wTei♂.2-3d 0.934971 0.141331 6.615 3.70e-11 ***

un♀ x wTei♂.5-6d 0.828557 0.139704 5.931 3.01e-09 ***

un♀ x wTei♂.9-10d 0.356023 0.141602 2.514 0.0119 *

wSuz♀ x wTei♂.2-3d 0.764682 0.143837 5.316 1.06e-07 ***

wSuz♀ x wTei♂.5-6d 0.970647 0.144017 6.740 1.59e-11 ***

wSuz♀x wTei♂.9-10d 0.301771 0.137714 2.191 0.0284 *

---

Signif. codes: 0 ‘***’ 0.001 ‘**’ 0.01 ‘*’ 0.05 ‘.’ 0.1 ‘ ’ 1

> library(blmeco)

> dispersion_glmer(m2)

[1] 1.022499

###group comparisons for the wTei line###

> library(multcomp)

> multiple_test2=summary(glht(m2,mcp(male_age_wTei="Tukey")))

> multiple_test2

Simultaneous Tests for General Linear Hypotheses

Multiple Comparisons of Means: Tukey Contrasts

Fit: glmer(formula = cbind(eggs, hatched) ~ male_age_wTei + (1 | batch2:rep2),

data = male4, family = "binomial")

Linear Hypotheses:

Estimate Std. Error z value Pr(>|z|)

un♀ x un♂.5-6d - un♀ x un♂.2-3d == 0 -9.008e-02 1.344e-01 -0.670 1.0000

un♀ x un♂.9-10d - un♀ x un♂.2-3d == 0 -1.964e-03 1.356e-01 -0.014 1.0000

wSuz♀ x wSuz♂.2-3d - un♀ x un♂.2-3d == 0 -8.998e-02 1.407e-01 -0.639 1.0000

wSuz♀ x wSuz♂.5-6d - un♀ x un♂.2-3d == 0 -1.208e-01 1.318e-01 -0.916 0.9990

wSuz♀ x wSuz♂.9-10d - un♀ x un♂.2-3d == 0 -5.913e-02 1.366e-01 -0.433 1.0000

un♀ x wTei♂.2-3d - un♀ x un♂.2-3d == 0 9.350e-01 1.413e-01 6.615 <0.01 ***

un♀ x wTei♂.5-6d - un♀ x un♂.2-3d == 0 8.286e-01 1.397e-01 5.931 <0.01 ***

un♀ x wTei♂.9-10d - un♀ x un♂.2-3d == 0 3.560e-01 1.416e-01 2.514 0.3306

wSuz♀ x wTei♂.2-3d - un♀ x un♂.2-3d == 0 7.647e-01 1.438e-01 5.316 <0.01 ***

wSuz♀ x wTei♂.5-6d - un♀ x un♂.2-3d == 0 9.706e-01 1.440e-01 6.740 <0.01 ***

wSuz♀ x wTei♂.9-10d - un♀ x un♂.2-3d == 0 3.018e-01 1.377e-01 2.191 0.5554

un♀ x un♂.9-10d - un♀ x un♂.5-6d == 0 8.812e-02 1.323e-01 0.666 1.0000

wSuz♀ x wSuz♂.2-3d - un♀ x un♂.5-6d == 0 9.833e-05 1.375e-01 0.001 1.0000

wSuz♀ x wSuz♂.5-6d - un♀ x un♂.5-6d == 0 -3.067e-02 1.283e-01 -0.239 1.0000

wSuz♀ x wSuz♂.9-10d - un♀ x un♂.5-6d == 0 3.095e-02 1.333e-01 0.232 1.0000

un♀ x wTei♂.2-3d - un♀ x un♂.5-6d == 0 1.025e+00 1.382e-01 7.418 <0.01 ***

un♀ x wTei♂.5-6d - un♀ x un♂.5-6d == 0 9.186e-01 1.365e-01 6.729 <0.01 ***

un♀ x wTei♂.9-10d - un♀ x un♂.5-6d == 0 4.461e-01 1.384e-01 3.223 0.0571 .

wSuz♀ x wTei♂.2-3d - un♀ x un♂.5-6d == 0 8.548e-01 1.406e-01 6.078 <0.01 ***

wSuz♀ x wTei♂.5-6d - un♀ x un♂.5-6d == 0 1.061e+00 1.409e-01 7.529 <0.01 ***

wSuz♀ x wTei♂.9-10d - un♀ x un♂.5-6d == 0 3.919e-01 1.344e-01 2.915 0.1346

wSuz♀ x wSuz♂.2-3d - un♀ x un♂.9-10d == 0 -8.802e-02 1.387e-01 -0.635 1.0000

wSuz♀ x wSuz♂.5-6d - un♀ x un♂.9-10d == 0 -1.188e-01 1.296e-01 -0.917 0.9990

wSuz♀ x wSuz♂.9-10d - un♀ x un♂.9-10d == 0 -5.717e-02 1.345e-01 -0.425 1.0000

un♀ x wTei♂.2-3d - un♀ x un♂.9-10d == 0 9.369e-01 1.392e-01 6.731 <0.01 ***

un♀ x wTei♂.5-6d - un♀ x un♂.9-10d == 0 8.305e-01 1.376e-01 6.038 <0.01 ***

un♀ x wTei♂.9-10d - un♀ x un♂.9-10d == 0 3.580e-01 1.396e-01 2.565 0.2982

wSuz♀ x wTei♂.2-3d - un♀ x un♂.9-10d == 0 7.666e-01 1.419e-01 5.404 <0.01 ***

wSuz♀ x wTei♂.5-6d - un♀ x un♂.9-10d == 0 9.726e-01 1.420e-01 6.850 <0.01 ***

wSuz♀ x wTei♂.9-10d - un♀ x un♂.9-10d == 0 3.037e-01 1.356e-01 2.240 0.5182

wSuz♀ x wSuz♂.5-6d - wSuz♀ x wSuz♂.2-3d == 0 -3.077e-02 1.349e-01 -0.228 1.0000

wSuz♀ x wSuz♂.9-10d - wSuz♀ x wSuz♂.2-3d == 0 3.085e-02 1.397e-01 0.221 1.0000

un♀ x wTei♂.2-3d - wSuz♀ x wSuz♂.2-3d == 0 1.025e+00 1.443e-01 7.105 <0.01 ***

un♀ x wTei♂.5-6d - wSuz♀ x wSuz♂.2-3d == 0 9.185e-01 1.427e-01 6.439 <0.01 ***

un♀ x wTei♂.9-10d - wSuz♀ x wSuz♂.2-3d == 0 4.460e-01 1.445e-01 3.086 0.0848 .

wSuz♀ x wTei♂.2-3d - wSuz♀ x wSuz♂.2-3d == 0 8.547e-01 1.467e-01 5.825 <0.01 ***

wSuz♀ x wTei♂.5-6d - wSuz♀ x wSuz♂.2-3d == 0 1.061e+00 1.469e-01 7.220 <0.01 ***

wSuz♀ x wTei♂.9-10d - wSuz♀ x wSuz♂.2-3d == 0 3.918e-01 1.407e-01 2.784 0.1868

wSuz♀ x wSuz♂.9-10d - wSuz♀ x wSuz♂.5-6d == 0 6.162e-02 1.306e-01 0.472 1.0000

un♀ x wTei♂.2-3d - wSuz♀ x wSuz♂.5-6d == 0 1.056e+00 1.356e-01 7.787 <0.01 ***

un♀ x wTei♂.5-6d - wSuz♀ x wSuz♂.5-6d == 0 9.493e-01 1.339e-01 7.091 <0.01 ***

un♀ x wTei♂.9-10d - wSuz♀ x wSuz♂.5-6d == 0 4.768e-01 1.358e-01 3.510 0.0228 *

wSuz♀ x wTei♂.2-3d - wSuz♀ x wSuz♂.5-6d == 0 8.854e-01 1.381e-01 6.409 <0.01 ***

wSuz♀ x wTei♂.5-6d - wSuz♀ x wSuz♂.5-6d == 0 1.091e+00 1.384e-01 7.889 <0.01 ***

wSuz♀ x wTei♂.9-10d - wSuz♀ x wSuz♂.5-6d == 0 4.225e-01 1.318e-01 3.206 0.0595 .

un♀ x wTei♂.2-3d - wSuz♀ x wSuz♂.9-10d == 0 9.941e-01 1.402e-01 7.088 <0.01 ***

un♀ x wTei♂.5-6d - wSuz♀ x wSuz♂.9-10d == 0 8.877e-01 1.386e-01 6.404 <0.01 ***

un♀ x wTei♂.9-10d - wSuz♀ x wSuz♂.9-10d == 0 4.152e-01 1.405e-01 2.954 0.1215

wSuz♀ x wTei♂.2-3d - wSuz♀ x wSuz♂.9-10d == 0 8.238e-01 1.428e-01 5.769 <0.01 ***

wSuz♀ x wTei♂.5-6d - wSuz♀ x wSuz♂.9-10d == 0 1.030e+00 1.430e-01 7.203 <0.01 ***

wSuz♀ x wTei♂.9-10d - wSuz♀ x wSuz♂.9-10d == 0 3.609e-01 1.366e-01 2.642 0.2558

un♀ x wTei♂.5-6d - un♀ x wTei♂.2-3d == 0 -1.064e-01 1.432e-01 -0.743 0.9999

un♀ x wTei♂.9-10d - un♀ x wTei♂.2-3d == 0 -5.789e-01 1.451e-01 -3.989 <0.01 **

wSuz♀ x wTei♂.2-3d - un♀ x wTei♂.2-3d == 0 -1.703e-01 1.474e-01 -1.155 0.9920

wSuz♀ x wTei♂.5-6d - un♀ x wTei♂.2-3d == 0 3.568e-02 1.475e-01 0.242 1.0000

wSuz♀ x wTei♂.9-10d - un♀ x wTei♂.2-3d == 0 -6.332e-01 1.413e-01 -4.481 <0.01 ***

un♀ x wTei♂.9-10d - un♀ x wTei♂.5-6d == 0 -4.725e-01 1.436e-01 -3.292 0.0464 *

wSuz♀ x wTei♂.2-3d - un♀ x wTei♂.5-6d == 0 -6.388e-02 1.458e-01 -0.438 1.0000

wSuz♀ x wTei♂.5-6d - un♀ x wTei♂.5-6d == 0 1.421e-01 1.459e-01 0.974 0.9982

wSuz♀ x wTei♂.9-10d - un♀ x wTei♂.5-6d == 0 -5.268e-01 1.397e-01 -3.772 <0.01 **

wSuz♀ x wTei♂.2-3d - un♀ x wTei♂.9-10d == 0 4.087e-01 1.475e-01 2.770 0.1919

wSuz♀ x wTei♂.5-6d - un♀ x wTei♂.9-10d == 0 6.146e-01 1.477e-01 4.160 <0.01 **

wSuz♀ x wTei♂.9-10d - un♀ x wTei♂.9-10d == 0 -5.425e-02 1.416e-01 -0.383 1.0000

wSuz♀ x wTei♂.5-6d - wSuz♀ x wTei♂.2-3d == 0 2.060e-01 1.499e-01 1.374 0.9683

wSuz♀ x wTei♂.9-10d - wSuz♀ x wTei♂.2-3d == 0 -4.629e-01 1.439e-01 -3.218 0.0577 .

wSuz♀ x wTei♂.9-10d - wSuz♀ x wTei♂.5-6d == 0 -6.689e-01 1.440e-01 -4.645 <0.01 ***

---

Signif. codes: 0 ‘***’ 0.001 ‘**’ 0.01 ‘*’ 0.05 ‘.’ 0.1 ‘ ’ 1

(Adjusted p values reported -- single-step method)

> library(emmeans)

> library(lsmeans)

> d <- emmeans(m2, "male_age_wTei", type= "response")

> cld(d)

male_age_wTei prob SE df asymp.LCL asymp.UCL .group

wSuz♀ x wSuz♂.5-6d 0.521 0.0222 Inf 0.478 0.564 1

un♀ x un♂.5-6d 0.529 0.0231 Inf 0.484 0.574 12

wSuz♀ x wSuz♂.2-3d 0.529 0.0253 Inf 0.479 0.578 12

wSuz♀ x wSuz♂.9-10d 0.537 0.0238 Inf 0.490 0.583 12

un♀ x un♂.9-10d 0.551 0.0233 Inf 0.505 0.596 12

un♀ x un♂.2-3d 0.551 0.0241 Inf 0.504 0.598 12

wSuz♀ x wTei♂.9-10d 0.624 0.0228 Inf 0.579 0.668 123

un♀ x wTei♂.9-10d 0.637 0.0238 Inf 0.589 0.682 23

wSuz♀ x wTei♂.2-3d 0.725 0.0211 Inf 0.682 0.765 34

un♀ x wTei♂.5-6d 0.738 0.0194 Inf 0.698 0.774 4

un♀ x wTei♂.2-3d 0.758 0.0188 Inf 0.719 0.793 4

wSuz♀ x wTei♂.5-6d 0.764 0.0191 Inf 0.725 0.800 4

Confidence level used: 0.95

Intervals are back-transformed from the logit scale

P value adjustment: tukey method for comparing a family of 12 estimates

Tests are performed on the log odds ratio scale

significance level used: alpha = 0.05

**###Irradiation of *Wolbachia*-infected adults**

###summary statistics for *w*Ha females###

> library(dplyr)

> group_by(whaf, inter2) %>%

+ summarise(

+ count = n(),

+ mean = mean(hatch_per, na.rm = TRUE),

+ sd = sd(hatch_per, na.rm = TRUE),

+ median = median(hatch_per, na.rm = TRUE),

+ IQR = IQR(hatch_per, na.rm = TRUE))

# A tibble: 5 x 6

inter2 count mean sd median IQR

*<fct>* *<int>* *<dbl>* *<dbl>* *<dbl>* *<dbl>*

1 0Gy.wHa 6 93.8 7.00 96 0.75

2 0Gy.wSuz 6 88.4 9.77 91.2 8.87

3 45Gy.wHa 3 0 0 0 0

4 60Gy.wHa 6 0 0 0 0

5 90Gy.wHa 3 0 0 0 0

> library(lme4)

> m2 <- glm(cbind(hatched, eggs)~inter2, data = whaf, family = "binomial")

> summary(m2)

Call:

glm(formula = cbind(hatched, eggs) ~ inter2, family = "binomial",

data = whaf)

Deviance Residuals:

Min 1Q Median 3Q Max

-1.4172 0.0000 0.0000 0.1598 0.7279

Coefficients: (2 not defined because of singularities)

Estimate Std. Error z value Pr(>|z|)

(Intercept) -0.06365 0.05868 -1.085 0.278

inter20Gy.wSuz -0.06005 0.08389 -0.716 0.474

inter245Gy.wHa -20.14436 6051.88957 -0.003 0.997

inter260Gy.wHa NA NA NA NA

inter290Gy.wHa NA NA NA NA

(Dispersion parameter for binomial family taken to be 1)

Null deviance: 12.7120 on 13 degrees of freedom

Residual deviance: 4.4414 on 11 degrees of freedom

AIC: 78.787

Number of Fisher Scoring iterations: 18

> kruskal.test(hatch_per~inter2, data = whaf)

Kruskal-Wallis rank sum test

data: hatch_per by inter2

Kruskal-Wallis chi-squared = 20.334, df = 4, p-value = 0.0004289

> dunn.test(x=whaf$hatch_per, g=whaf$inter2, method=p.adjustment.methods)

Kruskal-Wallis rank sum test

data: x and group

Kruskal-Wallis chi-squared = 20.3344, df = 4, p-value = 0

Comparison of x by group

(No adjustment)

Col Mean-|

Row Mean | 0Gy.wHa 0Gy.wSuz 45Gy.wHa 60Gy.wHa

---------+--------------------------------------------

0Gy.wSuz | 0.742586

| 0.2289

|

45Gy.wHa | 2.871101 2.264782

| 0.0020* 0.0118*

|

60Gy.wHa | 3.516367 2.773780 0.000000

| 0.0002* 0.0028* 0.5000

|

90Gy.wHa | 2.871101 2.264782 0.000000 0.000000

| 0.0020* 0.0118* 0.5000 0.5000

alpha = 0.05

Reject Ho if p <= alpha/2

###summary statistics for *w*Tei females###

> library(dplyr)

> group_by(wteif, wteif$inter2) %>%

+ summarise(

+ count = n(),

+ mean = mean(hatch_per, na.rm = TRUE),

+ sd = sd(hatch_per, na.rm = TRUE),

+ median = median(hatch_per, na.rm = TRUE),

+ IQR = IQR(hatch_per, na.rm = TRUE))

# A tibble: 5 x 6

`wteif$inter2` count mean sd median IQR

*<fct>* *<int>* *<dbl>* *<dbl>* *<dbl>* *<dbl>*

1 0Gy.wTei 6 92.3 7.28 93.5 7

2 0Gy.wSuz 6 88.4 9.77 91.2 8.87

3 45Gy.wTei 3 0 0 0 0

4 60Gy.wTei 6 3.33 8.16 0 0

5 90Gy.wTei 3 0 0 0 0

> library(lme4)

> m1 <- glm(cbind(nb_eggs, nb_hatched)~inter2, data = wteif, family = "binomial")

> summary(m1)

Call:

glm(formula = cbind(nb_eggs, nb_hatched) ~ inter2, family = "binomial",

data = wteif)

Deviance Residuals:

Min 1Q Median 3Q Max

-0.72786 -0.19823 0.00000 0.04411 1.41717

Coefficients:

Estimate Std. Error z value Pr(>|z|)

(Intercept) 7.976e-02 5.892e-02 1.354 0.176

inter20Gy.wSuz 4.394e-02 8.406e-02 0.523 0.601

inter245Gy.wTei 1.949e+01 1.075e+04 0.002 0.999

inter260Gy.wTei 1.712e+00 1.082e+00 1.583 0.114

inter290Gy.wTei 1.949e+01 1.075e+04 0.002 0.999

(Dispersion parameter for binomial family taken to be 1)

Null deviance: 11.1939 on 15 degrees of freedom

Residual deviance: 4.8909 on 11 degrees of freedom

AIC: 85.009

Number of Fisher Scoring iterations: 18

> kruskal.test(hatch_per~inter2, data = wteif)

Kruskal-Wallis rank sum test

data: hatch_per by inter2

Kruskal-Wallis chi-squared = 19.338, df = 4, p-value = 0.0006743

> dunn.test(x=wteif$hatch_per, g=wteif$inter2, method=p.adjustment.methods)

Kruskal-Wallis rank sum test

data: x and group

Kruskal-Wallis chi-squared = 19.3385, df = 4, p-value = 0

Comparison of x by group

(No adjustment)

Col Mean-|

Row Mean | 0Gy.wSuz 0Gy.wTei 45Gy.wTe 60Gy.wTe

---------+--------------------------------------------

0Gy.wTei | -0.343850

| 0.3655

|

45Gy.wTe | 2.491678 2.772430

| 0.0064* 0.0028*

|

60Gy.wTe | 2.793782 3.137632 -0.210564

| 0.0026* 0.0009* 0.4166

|

90Gy.wTe | 2.491678 2.772430 0.000000 0.210564

| 0.0064* 0.0028* 0.5000 0.4166

alpha = 0.05

Reject Ho if p <= alpha/2

###summary statistics for *w*Ha males###

> library(dplyr)

> group_by(wham, inter2) %>%

+ summarise(

+ count = n(),

+ mean = mean(hatch_per, na.rm = TRUE),

+ sd = sd(hatch_per, na.rm = TRUE),

+ median = median(hatch_per, na.rm = TRUE),

+ IQR = IQR(hatch_per, na.rm = TRUE))

# A tibble: 5 x 6

inter2 count mean sd median IQR

*<fct>* *<int>* *<dbl>* *<dbl>* *<dbl>* *<dbl>*

1 0Gy.wHa 6 93.8 7.00 96 0.75

2 0Gy.wSuz 6 88.4 9.77 91.2 8.87

3 45Gy.wHa 3 0.117 0.203 0 0.176

4 60Gy.wHa 6 0.333 0.816 0 0

5 90Gy.wHa 3 0.333 0.577 0 0.5

> library(lme4)

> m1 <- glm(cbind(eggs, hatched)~inter2, data = wham, family = "binomial")

> summary(m1)

Call:

glm(formula = cbind(eggs, hatched) ~ inter2, family = "binomial",

data = wham)

Deviance Residuals:

Min 1Q Median 3Q Max

-2.3029 -0.3403 0.1281 0.8358 1.4172

Coefficients:

Estimate Std. Error z value Pr(>|z|)

(Intercept) 0.06365 0.05868 1.085 0.278

inter20Gy.wSuz 0.06005 0.08389 0.716 0.474

inter245Gy.wHa 6.59820 1.00236 6.583 4.62e-11 ***

inter260Gy.wHa 6.07408 0.71030 8.551 < 2e-16 ***

inter290Gy.wHa 5.64013 1.00338 5.621 1.90e-08 ***

---

Signif. codes: 0 ‘***’ 0.001 ‘**’ 0.01 ‘*’ 0.05 ‘.’ 0.1 ‘ ’ 1

(Dispersion parameter for binomial family taken to be 1)

Null deviance: 1671.157 on 23 degrees of freedom

Residual deviance: 17.523 on 19 degrees of freedom

AIC: 102.45

Number of Fisher Scoring iterations: 6

> kruskal.test(hatch_per~inter2, data = wham)

Kruskal-Wallis rank sum test

data: hatch_per by inter2

Kruskal-Wallis chi-squared = 18.814, df = 4, p-value = 0.0008548

> dunn.test(x=wham$hatch_per, g=wham$inter2, method=p.adjustment.methods)

Kruskal-Wallis rank sum test

data: x and group

Kruskal-Wallis chi-squared = 18.8142, df = 4, p-value = 0

Comparison of x by group

(No adjustment)

Col Mean-|

Row Mean | 0Gy.wHa 0Gy.wSuz 45Gy.wHa 60Gy.wHa

---------+--------------------------------------------

0Gy.wSuz | 0.713686

| 0.2377

|

45Gy.wHa | 2.725085 2.142362

| 0.0032* 0.0161*

|

60Gy.wHa | 3.463479 2.749792 0.102833

| 0.0003* 0.0030* 0.4590

|

90Gy.wHa | 2.656530 2.073807 -0.059370 -0.171389

| 0.0039* 0.0190* 0.4763 0.4320

alpha = 0.05

Reject Ho if p <= alpha/2

###summary statistics for *w*Tei males###

> library(dplyr)

> group_by(wteim, wteim$inter2) %>%

+ summarise(

+ count = n(),

+ mean = mean(hatch_per, na.rm = TRUE),

+ sd = sd(hatch_per, na.rm = TRUE),

+ median = median(hatch_per, na.rm = TRUE),

+ IQR = IQR(hatch_per, na.rm = TRUE))

# A tibble: 5 x 6

`wteim$inter2` count mean sd median IQR

*<fct>* *<int>* *<dbl>* *<dbl>* *<dbl>* *<dbl>*

1 0Gy.wTei 6 92.3 7.28 93.5 7

2 0Gy.wSuz 6 88.4 9.77 91.2 8.87

3 45Gy.wTei 3 1.24 0.563 1.39 0.547

4 60Gy.wTei 6 2.27 1.54 2.63 2.06

5 90Gy.wTei 3 1 1 1 1

> library(lme4)

> m1 <- glm(cbind(eggs, hatched)~inter2, data = wteim, family = "binomial")

> summary(m1)

Call:

glm(formula = cbind(eggs, hatched) ~ inter2, family = "binomial",

data = wteim)

Deviance Residuals:

Min 1Q Median 3Q Max

-0.9385 -0.6222 -0.2150 0.5244 2.1714

Coefficients:

Estimate Std. Error z value Pr(>|z|)

(Intercept) 0.07976 0.05892 1.354 0.176

inter20Gy.wSuz 0.04394 0.08406 0.523 0.601

inter245Gy.wTei 4.42461 0.25089 17.636 < 2e-16 ***

inter260Gy.wTei 3.65600 0.21077 17.346 < 2e-16 ***

inter290Gy.wTei 4.52541 0.58321 7.759 8.53e-15 ***

---

Signif. codes: 0 ‘***’ 0.001 ‘**’ 0.01 ‘*’ 0.05 ‘.’ 0.1 ‘ ’ 1

(Dispersion parameter for binomial family taken to be 1)

Null deviance: 1843.892 on 23 degrees of freedom

Residual deviance: 17.769 on 19 degrees of freedom

AIC: 127.38

Number of Fisher Scoring iterations: 5

> kruskal.test(hatch_per~inter2, data = wteim)

Kruskal-Wallis rank sum test

data: hatch_per by inter2

Kruskal-Wallis chi-squared = 17.995, df = 4, p-value = 0.001237

> dunn.test(x=wteim$hatch_per, g=wteim$inter2, method=p.adjustment.methods)

Kruskal-Wallis rank sum test

data: x and group

Kruskal-Wallis chi-squared = 17.9948, df = 4, p-value = 0

Comparison of x by group

(No adjustment)

Col Mean-|

Row Mean | 0Gy.wSuz 0Gy.wTei 45Gy.wTe 60Gy.wTe

---------+--------------------------------------------

0Gy.wTei | -0.327096

| 0.3718

|

45Gy.wTe | 2.503813 2.770886

| 0.0061* 0.0028*

|

60Gy.wTe | 2.412338 2.739435 -0.534146

| 0.0079* 0.0031* 0.2966

|

90Gy.wTe | 2.637349 2.904423 0.115646 0.667683

| 0.0042* 0.0018* 0.4540 0.2522

alpha = 0.05

Reject Ho if p <= alpha/2

**###Quality control tests at 45Gy**

**### Emergence rate at 0 and 45Gy**

###summary statistics###

> library(dplyr)

> group_by(emer, emer$emergence) %>%

+ summarise(

+ count = n(),

+ mean = mean(emer_rate, na.rm = TRUE),

+ sd = sd(emer_rate, na.rm = TRUE),

+ median = median(emer_rate, na.rm = TRUE),

+ IQR = IQR(emer_rate, na.rm = TRUE))

# A tibble: 5 x 6

`emer$emergence` count mean sd median IQR

*<fct>* *<int>* *<dbl>* *<dbl>* *<dbl>* *<dbl>*

1 45Gy.wHa 5 70.7 19.2 73.3 0

2 control.wHa 5 44 16.7 46.7 6.67

3 control.wSuz 5 69.3 10.1 66.7 0

4 45Gy.wTei 5 52 5.58 53.3 6.67

5 control.wTei 5 68 14.5 60 6.67

> kruskal.test(emerged/pupae ~ emergence, data = emer)

Kruskal-Wallis rank sum test

data: emerged/pupae by emergence

Kruskal-Wallis chi-squared = 11.21, df = 4, p-value = 0.0243

> library(lme4)

> m1 <- glmer(emerged2~emergence1+(1|replicate:pupae), data = emer2, family = "binomial")

> summary(m1)

Generalized linear mixed model fit by maximum likelihood (Laplace Approximation) ['glmerMod']

Family: binomial ( logit )

Formula: emerged2 ~ emergence1 + (1 | replicate:pupae)

Data: emer2

AIC BIC logLik deviance df.resid

496.0 519.6 -242.0 484.0 369

Scaled residuals:

Min 1Q Median 3Q Max

-1.5521 -1.0408 0.6443 0.6860 1.1281

Random effects:

Groups Name Variance Std.Dev.

replicate:pupae (Intercept) 1.001e-08 0.0001001

Number of obs: 375, groups: replicate:pupae, 375

Fixed effects:

Estimate Std. Error z value Pr(>|z|)

(Intercept) 0.8792 0.2536 3.467 0.000527 ***

emergence1control.wHa -1.1204 0.3442 -3.256 0.001132 **

emergence1control.wSuz -0.0635 0.3564 -0.178 0.858594

emergence145Gy.wTei -0.7992 0.3431 -2.329 0.019852 *

emergence1control.wTei -0.1255 0.3544 -0.354 0.723291

---

Signif. codes: 0 ‘***’ 0.001 ‘**’ 0.01 ‘*’ 0.05 ‘.’ 0.1 ‘ ’ 1

Correlation of Fixed Effects:

(Intr) emr1.H emr1.S e145G.

emrgnc1cn.H -0.737

emrgnc1cn.S -0.712 0.524

emrgn145G.T -0.739 0.545 0.526

emrgnc1cn.T -0.716 0.527 0.509 0.529

> library(blmeco)

> dispersion_glmer(m1) #result is not >1.4 so NO overdispersion problem

[1] 1.136074

> library(multcomp)

> multiple_test=summary(glht(m1,mcp(emergence1="Tukey")))

> multiple_test

Simultaneous Tests for General Linear Hypotheses

Multiple Comparisons of Means: Tukey Contrasts

Fit: glmer(formula = emerged2 ~ emergence1 + (1 | replicate:pupae),

data = emer2, family = "binomial")

Linear Hypotheses:

Estimate Std. Error z value Pr(>|z|)

control.wHa - 45Gy.wHa == 0 -1.12041 0.34415 -3.256 0.00994 **

control.wSuz - 45Gy.wHa == 0 -0.06350 0.35641 -0.178 0.99977

45Gy.wTei - 45Gy.wHa == 0 -0.79921 0.34313 -2.329 0.13545

control.wTei - 45Gy.wHa == 0 -0.12548 0.35439 -0.354 0.99664

control.wSuz - control.wHa == 0 1.05691 0.34180 3.092 0.01693 *

45Gy.wTei - control.wHa == 0 0.32120 0.32792 0.980 0.86449

control.wTei - control.wHa == 0 0.99493 0.33970 2.929 0.02802 *

45Gy.wTei - control.wSuz == 0 -0.73571 0.34078 -2.159 0.19535

control.wTei - control.wSuz == 0 -0.06198 0.35211 -0.176 0.99979

control.wTei - 45Gy.wTei == 0 0.67373 0.33867 1.989 0.27089

---

Signif. codes: 0 ‘***’ 0.001 ‘**’ 0.01 ‘*’ 0.05 ‘.’ 0.1 ‘ ’ 1

(Adjusted p values reported -- single-step method)

**### Female longevity at 0 and 45Gy**

> library(survival)

> mfit <- survfit(Surv(time=longf$lifespan, event=longf$event) ~ longevity, data = longf)

> mfit

Call: survfit(formula = Surv(time = longf$lifespan, event = longf$event) ~

longevity, data = longf)

n events median 0.95LCL 0.95UCL

longevity=wHa.45Gy 45 28 58 48 NA

longevity=wTei.45Gy 45 24 49 40 NA

longevity=wHa.control 44 42 48 38 58

longevity=wTei.control 44 24 58 48 NA

> summary(mfit)

Call: survfit(formula = Surv(time = longf$lifespan, event = longf$event) ~

longevity, data = longf)

longevity=wHa.45Gy

time n.risk n.event survival std.err lower 95% CI upper 95% CI

26 45 2 0.956 0.0307 0.897 1.000

34 43 6 0.822 0.0570 0.718 0.942

38 37 4 0.733 0.0659 0.615 0.875

45 33 4 0.644 0.0714 0.519 0.801

48 29 4 0.556 0.0741 0.428 0.721

55 25 2 0.511 0.0745 0.384 0.680

58 23 6 0.378 0.0723 0.260 0.550

longevity=wTei.45Gy

time n.risk n.event survival std.err lower 95% CI upper 95% CI

2 45 1 0.978 0.0220 0.936 1.000

13 44 1 0.956 0.0307 0.897 1.000

16 43 1 0.933 0.0372 0.863 1.000

25 42 1 0.911 0.0424 0.832 0.998

39 41 11 0.667 0.0703 0.542 0.820

40 30 7 0.511 0.0745 0.384 0.680

49 23 1 0.489 0.0745 0.363 0.659

50 22 1 0.467 0.0744 0.341 0.638

longevity=wHa.control

time n.risk n.event survival std.err lower 95% CI upper 95% CI

3 44 1 0.9773 0.0225 0.9342 1.000

5 43 1 0.9545 0.0314 0.8949 1.000

6 42 1 0.9318 0.0380 0.8602 1.000

8 41 1 0.9091 0.0433 0.8280 0.998

19 40 1 0.8864 0.0478 0.7974 0.985

21 39 1 0.8636 0.0517 0.7680 0.971

22 38 1 0.8409 0.0551 0.7395 0.956

30 37 2 0.7955 0.0608 0.6848 0.924

33 35 2 0.7500 0.0653 0.6324 0.890

35 33 2 0.7045 0.0688 0.5818 0.853

36 31 1 0.6818 0.0702 0.5572 0.834

37 30 2 0.6364 0.0725 0.5090 0.796

38 28 3 0.5682 0.0747 0.4392 0.735

48 25 9 0.3636 0.0725 0.2460 0.538

58 16 14 0.0455 0.0314 0.0117 0.176

longevity=wTei.control

time n.risk n.event survival std.err lower 95% CI upper 95% CI

8 44 1 0.977 0.0225 0.934 1.000

23 43 1 0.955 0.0314 0.895 1.000

31 42 1 0.932 0.0380 0.860 1.000

32 41 3 0.864 0.0517 0.768 0.971

35 38 1 0.841 0.0551 0.739 0.956

48 37 11 0.591 0.0741 0.462 0.756

58 26 6 0.455 0.0751 0.329 0.628

> mdiff.comp<- survdiff(Surv(time=longf$lifespan, event=longf$event)~ longevity , data = longf)

> mdiff.comp

Call:

survdiff(formula = Surv(time = longf$lifespan, event = longf$event) ~

longevity, data = longf)

N Observed Expected (O-E)^2/E (O-E)^2/V

longevity=wHa.45Gy 45 28 30.9 0.272 0.421

longevity=wTei.45Gy 45 24 29.0 0.870 1.315

longevity=wHa.control 44 42 24.3 12.880 18.454

longevity=wTei.control 44 24 33.8 2.826 4.555

Chisq= 19.2 on 3 degrees of freedom, p= 2e-04

**### Male longevity at 0 and 45Gy**

> library(survival)

> mfit <- survfit(Surv(time=longm$lifespan, event=longm$event) ~ longevity, data = longm)

> mfit

Call: survfit(formula = Surv(time = longm$lifespan, event = longm$event) ~

longevity, data = longm)

n events median 0.95LCL 0.95UCL

longevity=wHa.45Gy 45 26 58 58 NA

longevity=wTei.45Gy 44 3 NA NA NA

longevity=wHa.control 45 23 58 58 NA

longevity=wTei.control 42 21 58 34 NA

> summary(mfit)

Call: survfit(formula = Surv(time = longm$lifespan, event = longm$event) ~

longevity, data = longm)

longevity=wHa.45Gy

time n.risk n.event survival std.err lower 95% CI upper 95% CI

0 45 1 0.978 0.0220 0.936 1.000

9 44 1 0.956 0.0307 0.897 1.000

24 43 1 0.933 0.0372 0.863 1.000

28 42 2 0.889 0.0468 0.802 0.986

32 40 1 0.867 0.0507 0.773 0.972

38 39 3 0.800 0.0596 0.691 0.926

39 36 4 0.711 0.0676 0.590 0.857

48 32 3 0.644 0.0714 0.519 0.801

58 20 10 0.322 0.0804 0.198 0.525

longevity=wTei.45Gy

time n.risk n.event survival std.err lower 95% CI upper 95% CI

2 44 1 0.977 0.0225 0.934 1

4 43 1 0.955 0.0314 0.895 1

19 42 1 0.932 0.0380 0.860 1

longevity=wHa.control

time n.risk n.event survival std.err lower 95% CI upper 95% CI

6 45 1 0.978 0.0220 0.936 1.000

24 43 1 0.955 0.0311 0.896 1.000

25 42 1 0.932 0.0377 0.861 1.000

30 41 1 0.910 0.0431 0.829 0.998

31 40 1 0.887 0.0477 0.798 0.985

38 39 1 0.864 0.0516 0.769 0.971

48 38 5 0.750 0.0652 0.633 0.890

58 33 12 0.478 0.0753 0.351 0.650

longevity=wTei.control

time n.risk n.event survival std.err lower 95% CI upper 95% CI

5 42 2 0.952 0.0329 0.890 1.000

6 40 2 0.905 0.0453 0.820 0.998

8 38 1 0.881 0.0500 0.788 0.985

14 37 1 0.857 0.0540 0.758 0.970

15 36 1 0.833 0.0575 0.728 0.954

17 35 1 0.810 0.0606 0.699 0.937

18 34 1 0.786 0.0633 0.671 0.920

24 33 2 0.738 0.0678 0.616 0.884

25 31 1 0.714 0.0697 0.590 0.865

31 30 1 0.690 0.0713 0.564 0.845

34 29 3 0.619 0.0749 0.488 0.785

35 26 1 0.595 0.0757 0.464 0.764

38 25 1 0.571 0.0764 0.440 0.743

48 24 1 0.548 0.0768 0.416 0.721

58 23 2 0.500 0.0772 0.370 0.677

**### Flight ability at 0 and 45Gy**

###summary statistics for the *w*Ha line###

> library(dplyr)

> group_by(fly3, trt2) %>%

+ summarise(

+ count = n(),

+ mean = mean(fliers_per, na.rm = TRUE),

+ sd = sd(fliers_per, na.rm = TRUE),

+ median = median(fliers_per, na.rm = TRUE),

+ IQR = IQR(fliers_per, na.rm = TRUE))

# A tibble: 2 x 6

trt2 count mean sd median IQR

*<fct>* *<int>* *<dbl>* *<dbl>* *<dbl>* *<dbl>*

1 45Gy 5 98.6 3.19 100 0

2 control 5 90.7 6.88 91.7 6.67

###summary statistics for the *w*Tei line###

> group_by(fly4, trt2) %>%

+ summarise(

+ count = n(),

+ mean = mean(fliers_per, na.rm = TRUE),

+ sd = sd(fliers_per, na.rm = TRUE),

+ median = median(fliers_per, na.rm = TRUE),

+ IQR = IQR(fliers_per, na.rm = TRUE))

# A tibble: 2 x 6

trt2 count mean sd median IQR

*<fct>* *<int>* *<dbl>* *<dbl>* *<dbl>* *<dbl>*

1 45Gy 5 78.5 10.3 73.3 14.3

2 control 5 82.1 4.09 83.3 7.14

###GLMM for the wHa and wTei lines###

> library(lme4)

> m1 <- glmer(fliers2~flying2+(1|rep2:emerged2), data = fly2, family = "binomial")

Warning message:

In checkConv(attr(opt, "derivs"), opt$par, ctrl = control$checkConv, :

Model failed to converge with max|grad| = 0.626504 (tol = 0.001, component 1)

> m3 <- update(m1, control=glmerControl(optimizer="bobyqa",

+ optCtrl=list(maxfun=2e5)))

> summary(m3)

Generalized linear mixed model fit by maximum likelihood (Laplace Approximation) ['glmerMod']

Family: binomial ( logit )

Formula: fliers2 ~ flying2 + (1 | rep2:emerged2)

Data: fly2

Control: glmerControl(optimizer = "bobyqa", optCtrl = list(maxfun = 2e+05))

AIC BIC logLik deviance df.resid

241.9 264.5 -114.9 229.9 316

Scaled residuals:

Min 1Q Median 3Q Max

-8.3066 0.1204 0.3136 0.4671 0.5222

Random effects:

Groups Name Variance Std.Dev.

rep2:emerged2 (Intercept) 4e-08 2e-04

Number of obs: 322, groups: rep2:emerged2, 322

Fixed effects:

Estimate Std. Error z value Pr(>|z|)

(Intercept) 4.234 1.007 4.203 2.63e-05 ***

flying2control.wHa -1.915 1.094 -1.750 0.08010 .

flying2control.wSuz -2.625 1.079 -2.432 0.01500 *

flying245Gy.wTei -2.935 1.048 -2.799 0.00512 **

flying2control.wTei -2.712 1.056 -2.567 0.01026 *

---

Signif. codes: 0 ‘***’ 0.001 ‘**’ 0.01 ‘*’ 0.05 ‘.’ 0.1 ‘ ’ 1

Correlation of Fixed Effects:

(Intr) fly2.H fly2.S f245G.

flyng2cnt.H -0.920

flyng2cnt.S -0.933 0.859

flyng245G.T -0.961 0.884 0.897

flyng2cnt.T -0.953 0.878 0.890 0.916

> library(blmeco)

> dispersion_glmer(m3)

[1] 0.8449028

> library(multcomp)

> multiple_test2=summary(glht(m3,mcp(flying2="Tukey")))

> multiple_test2 ###use these p-values

Simultaneous Tests for General Linear Hypotheses

Multiple Comparisons of Means: Tukey Contrasts

Fit: glmer(formula = fliers2 ~ flying2 + (1 | rep2:emerged2), data = fly2,

family = "binomial", control = glmerControl(optimizer = "bobyqa",

optCtrl = list(maxfun = 2e+05)))

Linear Hypotheses:

Estimate Std. Error z value Pr(>|z|)

control.wHa - 45Gy.wHa == 0 -1.91499 1.09423 -1.750 0.3824

control.wSuz - 45Gy.wHa == 0 -2.62467 1.07903 -2.432 0.0970 .

45Gy.wTei - 45Gy.wHa == 0 -2.93482 1.04842 -2.799 0.0368 *

control.wTei - 45Gy.wHa == 0 -2.71168 1.05633 -2.567 0.0691 .

control.wSuz - control.wHa == 0 -0.70968 0.57713 -1.230 0.7177

45Gy.wTei - control.wHa == 0 -1.01983 0.51764 -1.970 0.2625

control.wTei - control.wHa == 0 -0.79669 0.53347 -1.493 0.5462

45Gy.wTei - control.wSuz == 0 -0.31015 0.48462 -0.640 0.9657

control.wTei - control.wSuz == 0 -0.08701 0.50151 -0.173 0.9998

control.wTei - 45Gy.wTei == 0 0.22314 0.43170 0.517 0.9844

---

Signif. codes: 0 ‘***’ 0.001 ‘**’ 0.01 ‘*’ 0.05 ‘.’ 0.1 ‘ ’ 1

(Adjusted p values reported -- single-step method)

> cld(multiple_test2)

45Gy.wHa control.wHa control.wSuz 45Gy.wTei control.wTei

"b" "ab" "ab" "a" "ab"
